# The Fusion Protein rFlaA:Betv1 Modulates DC Responses by a p38-MAPK and COX2-Dependent Secretion of PGE_2_ from Epithelial Cells

**DOI:** 10.3390/cells10123415

**Published:** 2021-12-04

**Authors:** Yen-Ju Lin, Adam Flaczyk, Sonja Wolfheimer, Alexandra Goretzki, Annette Jamin, Andrea Wangorsch, Stefan Vieths, Stephan Scheurer, Stefan Schülke

**Affiliations:** 1 Molecular Allergology, Paul-Ehrlich-Institut, 63225 Langen, Germany; Yen-Ju.Lin@pei.de (Y.-J.L.); aflaczyk@mgh.harvard.edu (A.F.); Sonja.Wolfheimer@pei.de (S.W.); Alexandra.Goretzki@pei.de (A.G.); Annette.Jamin@pei.de (A.J.); Andrea.Wangorsch@pei.de (A.W.); Stefan.Vieths@pei.de (S.V.); Stephan.Scheurer@pei.de (S.S.); 2 Department of Anesthesia, Critical Care and Pain Medicine, Massachusetts General Hospital, Harvard Medical School, Boston, MA 02114, USA

**Keywords:** flagellin, Betv1, epithelial cells, vaccine, fusion protein

## Abstract

Developing new adjuvants/vaccines and better understanding their mode-of-action is an important task. To specifically improve birch pollen allergy treatment, we designed a fusion protein consisting of major birch pollen allergen Betv1 conjugated to the TLR5-ligand flagellin (rFlaA:Betv1). This study investigates the immune-modulatory effects of rFlaA:Betv1 on airway epithelial cells. LA-4 mouse lung epithelial cells were stimulated with rFlaA:Betv1 in the presence/absence of various inhibitors with cytokine- and chemokine secretion quantified by ELISA and activation of intracellular signaling cascades demonstrated by Western blot (WB). Either LA-4 cells or LA-4-derived supernatants were co-cultured with BALB/c bone marrow-derived myeloid dendritic cells (mDCs). Compared to equimolar amounts of flagellin and Betv1 provided as a mixture, rFlaA:Betv1 induced higher secretion of IL-6 and the chemokines CCL2 and CCL20 from LA-4 cells and a pronounced MAPK- and NF*κ*B-activation. Mechanistically, rFlaA:Betv1 was taken up more strongly and the induced cytokine production was inhibited by NF*κ*B-inhibitors, while ERK- and p38-MAPK-inhibitors only suppressed IL-6 and CCL2 secretion. In co-cultures of LA-4 cells with mDCs, rFlaA:Betv1-stimulated LA-4 cells p38-MAPK- and COX2-dependently secreted PGE_2_, which modulated DC responses by suppressing pro-inflammatory IL-12 and TNF-α secretion. Taken together, these results contribute to our understanding of the mechanisms underlying the strong immune-modulatory effects of flagellin-containing fusion proteins.

## 1. Introduction

The current SARS-CoV-2 pandemic has clearly demonstrated the need to develop both safe and efficacious vaccines. Protein-antigen-based vaccines that typically have a low immunogenicity often need to be adjuvanted in order to induce robust immune responses.

In this context, some “Toll”-like receptor (TLR) ligands with intrinsic immune-activating properties are attractive adjuvant candidates. They act as pathogen-associated molecular patterns (PAMPs) which bind conserved pattern recognition receptors (PRRs) on immune cells to induce strong immune responses.

Currently, monophosphporyl lipid A (MPLA), a modified TLR4 ligand derived from the lipopolysaccharide of *Salmonella minnesota* R595 is the only TLR ligand that is already used as an adjuvant in licensed vaccines. Here, several vaccines containing MPLA as a component of more complex adjuvant systems have been licensed or are undergoing phase III clinical trials. These include Fendrix^®^ (for the prevention of hepatitis B), Cervarix^®^ (human papillomavirus-16 and papillomavirus-18), RTS,S(R) (malaria), and the allergen product Pollinex^®^ Quattro (pollen allergies) [1,2,3].

The success of MPLA as a vaccine adjuvant clearly demonstrates the immune-modulating potential of TLR-ligands. However, other TLR-ligands were less successful in pre-clinical trials. For example, nucleic acid-based TLR-ligands, such as CpG (activating TLR9), R848 (TLR7/8), or Poly I:C (TLR3), are potent immune activators, but are limited in their clinical efficacy due to problems with both toxicity and stability in vivo [4].

So far, the TLR5 ligand flagellin, a bacterial motility protein that forms the main body of the bacterial flagellum [5], is the only purely protein-based TLR ligand. In addition to TLR5, flagellin activates intracellular NOD-like receptor 4 (NLRC4) resulting in inflammation and IL-1β production [6]. Consequently, flagellin has repeatedly demonstrated mucosal adjuvant properties resulting in the induction of protective immune responses [7,8,9].

Flagellin being a protein allows for the generation of recombinant flagellin antigen fusion proteins by recombinant DNA technology. Such flagellin-containing fusion proteins have been investigated as vaccine candidates in pre-clinical models for the prevention of influenza [10,11,12], poxvirus [13], West–Nile virus [14], tetanus [15], and *Pseudomonas* infections [16], as well as for the treatment of allergic diseases [17,18,19,20,21,22,23]. Moreover, fusion proteins containing *Salmonella* flagellin C and influenza antigens have been shown to be both safe and well-tolerated in clinical trials [24,25].

Mechanistically, TLR ligand:antigen fusion proteins effectively target the fused antigen to immune cells in vivo that express their respective TLR, resulting in more effective processing and simultaneous presentation of the enclosed antigen in the context of the TLR ligand-mediated immune cell activation [9,26].

We recently generated a fusion protein consisting of flagellin A from *Listeria monocytogenes* and the major birch pollen allergen Betv1 (rFlaA:Betv1) that was able to suppress allergic sensitization in vivo [22]. In vitro analyses in myeloid dendritic cells (mDCs) showed rFlaA:Betv1 to induce a pronounced mDC activation characterized by the secretion of both pro- (IL-6, IL-12, and TNF-α) and anti-inflammatory (IL-10) cytokines as well as pronounced surface expression of co-stimulatory molecules [22]. While the effects of flagellin antigen fusion proteins on DCs are well investigated, their effects on other cell types require further investigation.

Epithelial cells, which similarly express high levels of TLR5 [27] are the first line of defense against invading pathogens [28]. In addition to their function as part of the mechanical barrier, epithelial cells engage in the initiation and maintenance of immune responses [29,30]. Just as other immune cells, epithelial cells express pattern recognition receptors, become activated, and secrete both cytokines and chemokines to alert other immune cells to invading pathogens [28]. Therefore, epithelial cells play an integral part in the establishment of immune responses.

To our knowledge, the effects of fusion proteins on epithelial cells have been poorly studied. Maffía et al. showed that fusion of Cementoin to the secretory leukocyte proteinase inhibitor (SLPI) increased both SPLI’s preoteolytic stability and surface binding to TNF-α- or LPS-pretreated A549 alveolar epithelial cells [31]. Moreover, Savar et al. in 2014 generated a FimH/tFliC fusion protein consisting of a truncated form of flagellin C (FliC) from enteroaggregative *E. coli* fused to FimH, the tip adhesion on type 1 fimbria, from uropathogenic *E. coli* to create a vaccine candidate against urinary tract infections [32]. Despite showing that the truncated form of FliC alone could induce IL-8 in the HT-29 epithelial cell line [32], no experiments using this fusion protein on epithelial cells were performed [32].

Therefore, as far as we know, the effects of flagellin:antigen fusion proteins on epithelial cells and intracellular signal transduction are currently unknown.

In the present study, we analyzed the effects of rFlaA:Betv1 on the mouse lung epithelial cell line LA-4. Here, we show that rFlaA:Betv1 is taken up more readily than the mixture of both proteins and induces a MAPK- and NF*κ*B-dependent secretion of both cytokines and chemokines. Moreover, rFlaA:Betv1-stimulated LA-4 cells modulated cytokine secretion from BALB/c mDCs stimulated with rFlaA:Betv1. Supernatants derived from rFlaA:Betv1-stimulated LA-4 cells selectively suppressed rFlaA:Betv1-induced, mDC-derived IL-12 and TNF-α secretion. These immune-modulatory effects of LA-4 cells were shown to depend on the p38-MAPK- and cyclooxygenase-2 (COX2)-dependent production of prostaglandin E2 (PGE_2_).

## 2. Materials and Methods

### 2.1. Flow Cytometry

For characterisation of LA-4 cells, the following antibodies were used: PE-conjugated anti-mouse TLR5 (clone: 85B152.5, 1:100, Abcam, Cambridge, UK), PE-conjugated anti-mouse MHCI (clone: 31-1-2S, 1:100, eBiosciences, Frankfurt, Germany), AF488-conjugated anti-mouse Pan-cytokeratin (clone: AE1/AE3, 1:50, eBiosciences), AF488-conjugated anti-human mucin 1 (clone: SM3, 1:25, eBiosciences), APC-conjugated anti-mouse EpCAM (clone: G8.8, 1:50, eBiosciences), and APC-conjugated anti-mouse CEACAM1 (clone: CC1, 1:50, eBiosciences). Bone marrow-derived mDCs were characterized using anti-mouse pacific blue-conjugated CD11b (clone: M1/70.15, 1:50, Invitrogen, ThermoFisher Scientific, Dreieich, Germany), APC-conjugated CD11c (clone: HL3, dilution: 1:500, BD Bioscience), and PE-Cy5-conjugated B220 (clone: RA3-6B2, dilution: 1:100, BD Bioscience), anti-mouse FITC-conjugated CD3 antibody (clone: 145-2C11, 1:50, BD Biosciences), anti-mouse FITC-conjugated CD19 antibody (clone: 6D5, 1:50, Southern Biotech), and anti-mouse FITC-conjugated CD49b pan NK cell antibody (clone: Dx5, 1:100, BioLegend). Cells were analyzed by flow cytometry using a LSR II flow cytometer (BD Bioscience). Data were analyzed using FlowJo either V.7 or V.10 (Treestar Inc., Ashland, OR, USA).

### 2.2. Generation of Recombinant Proteins

Recombinant flagellin A from *Listeria monocytogenes* (rFlaA, Acc. No: NC_003210) was generated according to [19], and recombinant birch pollen allergen Betv1 (Acc. No: X15877.1) according to [33]. The fusion protein of rFlaA and rBetv1 (rFlaA:Betv1) was generated according to [22] by cDNA fusion using the cDNAs of both rFlaA and rBetv1 as templates. For the generation of rFlaA^*D1^ and rFlaA^*D1^:Betv1 mutants, amino acids at position 87–94 of the *Listeria monocytogenes* flagellin A D1 domain (QRMRQLAV) were substituted by PCR mutagenesis strategy (Q5^®^ Site-Directed Mutagenesis Kit, NEB, Frankfurt, Germany) with the corresponding sequence stretch from *Helicobacter pylori* flagellin (DTVKVKAT), which was reported to avoid TLR5 binding [34]. Protein expression and purification strategy of the mutants was performed according to [19]. Briefly, BL21 star DE3 cells (Invitrogen, Karlsruhe, Germany) transformed with the cloned mutant sequences in pET15b (Novagen, Darmstadt, Germany) were cultured in 4L of LB-medium supplemented with 50 mg/L of carbenicillin (Roth, Karlsruhe, Germany). Protein expression was induced by 1 mM IPTG at OD_600_ = 0.5, and cells were incubated for 20 h at 25 °C, 180 rpm. Subsequently, cells were harvested, lysed by sonication, and repeated freeze–thawing cycles. Inclusion bodies were solubilized with 50 mM phosphate buffer plus 6 M urea, and proteins were re-folded and purified with His GraviTrap columns (GE Healthcare, Freiburg, Germany). To further improve protein purity and remove endotoxins, a size exclusion chromatography using a HiLoad^®^ 26/600 Superdex^®^ 75 pg column (Cytiva, Freiburg (Breisgau), Germany) was performed. All proteins displayed a purity greater than 98%, the correct folding of secondary structure elements as determined by circular dichroism spectroscopy, and endotoxin contents of 1.14 pg/µg protein (rFlaA), <0.48 pg/µg protein (rBetv1), and 1.7 pg/µg protein (rFlaA:Betv1) <0.963 pg/µg protein (rFlaA^*D1^), and 2.7 pg/µg protein (rFlaA^*D1^:Betv1), respectively (data not shown).

### 2.3. Culture, Stimulation, Viability of LA-4 Cells

The mouse lung epithelial cell line LA-4 (ATCC CCL-196TM) was cultured in DMEM medium (Gibco, Karlsruhe, Germany), supplemented with 15% FCS (Sigma-Aldrich, Taufkirchen, Germany), 100 U/mL penicillin, 100 µg/mL streptomycin, and 1 mM L-glutamine. Cells were passaged every 3–4 days using 0.05% trypsin-EDTA. For stimulations, LA-4 cells were seeded overnight at 6.25 × 10^4^ cells/mL in 24-well plates (Thermo Scientific, Dreieich, Germany) and on the following morning stimulated with the indicated equimolar concentrations of either rFlaA + rBetv1, or rFlaA:Betv1 for either 2, 4, or 24 h. LPS (#L5886, Sigma-Aldrich) at 10 µg/mL served as a positive control.

### 2.4. ELISA

Cytokines in the supernatants were analyzed by ELISA using the following antibody combinations: IL-1β (capture antibody: anti-IL-1β monoclonal mouse antibody (#14-7061-85, eBioscience, Frankfurt, Germany, 1:500); plus detection antibody anti-IL-1β monoclonal mouse biotin-conjugated antibody (#13-7112-81, eBioscience, 1:500)), IL-6 (capture antibody: anti-IL-6 monoclonal mouse antibody (#14-7061-85, eBioscience, Frankfurt, Germany, 1:500); plus detection antibody anti-IL-6 monoclonal mouse biotin-conjugated antibody (#13-7062-85, eBioscience, 1:500)), TNF-α (capture antibody: anti-TNF-α monoclonal mouse antibody (#14-7325-85, eBioscience, 1:500); plus detection antibody anti-TNF-α monoclonal mouse biotin-conjugated antibody (#13-7326-85, eBioscience, 1:500), IL-12 p70 (capture antibody: anti-IL-12 monoclonal mouse antibody (#14-7122-85, eBioscience, 1:500); plus detection antibody: anti-IL-12 monoclonal mouse biotin-conjugated antibody (#MM121B, Invitrogen, 1:500). Following incubation with the respective detection antibodies, plates were incubated with 50 µL of diluted streptavidin horseradish peroxidase (#554066, eBioscience, 1:2000) for 30 min at room temperature. Detection was performed with 100 µL 3,3′,5,5′-tetramethylbenzidine (Carl Roth Chemikalien, Karlsruhe, Germany) and incubation for 3 to 5 min at room temperature. The reaction was stopped with 50 µL per well of 1 M sulfuric acid (Carl Roth Laborbedarf, Karlsruhe, Germany). Optical density was measured at 450 nm by SpectraMAX340PC (Molecular Devices, CA, USA). Levels of IL-10, CCL2, CCL20, and PGE_2_ were measured according to the manufacturer’s recommendations using either the Murine IL-10 standard ABTS ELISA development kit (Peprotech, Hamburg, Germany), mouse CCL2 ELISA development kit from Invitrogen (#900-T53), CCL20/MIP-3 alpha DuoSet (#DY760, R&D Systems, Wiesbaden-Nordenstadt, Germany), or the PGE_2_ ELISA kit (#KA4522, Abnova, Taipei, Taiwan), respectively.

### 2.5. Inhibitors

LA-4 cells were pre-incubated with the indicated amounts of either the mTOR inhibitor rapamycin (Invivogen, Toulouse, France), MAPK-inhibitors U0126 (MEK1/2 MAPK inhibitor, Cell Signaling Technologies, Leiden, The Netherlands), SP600125 (SAP/JNK MAPK inhibitor, Invivogen), SB-202190 (p38α/β MAPK inhibitor, Invivogen), the NF*κ*B- and MAPK-inhibitor dexamethasone (Invivogen), the IKK-β-inhibitor TPCA-1 (Abcam, Berlin, Germany), the inhibitor of actin polymerization cytochalasin A (Sigma-Aldrich), the inhibitor of endosomal acidification chloroquine (InvivoGen), or the COX2-inhibitor NS-398 (Sigma-Aldrich, Steinheim, Germany) for 90 min and subsequently stimulated with rFlaA:Betv1 for 24 h. For analyzing cell viability, LA-4 cells were treated as indicated and stained for dead cells using the fixable viability dye eFlour450 (#65-0865-14, eBioscience) and measured by FACS. Data were analyzed using FlowJo V.7 (Treestar Inc., Ashland, OR, USA) and GraphPad PRISM (GraphPad Software, San Diego, CA, USA).

### 2.6. Western Blot

For Western blot experiments, LA-4 cells were seeded overnight at 1.66 × 10^5^ cells/2 mL in 6-well plates (Thermo Scientific) with culture medium. On the next morning, cells were cultured for 3 h at 37 °C, 5% CO_2_ in DMEM supplemented with 2% FCS (Sigma-Aldrich) for starvation, and subsequently, stimulated with the indicated proteins in DMEM for 30 min, washed with ice-cold PBS, and lysed with 200 µL lysis buffer (62.5 mM Tris-HCl (pH 6.8), 2% *w*/*v* SDS, 10% glycerol, 50 mM DTT, 0.01% *w*/*v* bromophenol blue) for 10 min on ice. Target proteins in lysates were separated by SDS-polyacrylamide gel electrophoresis and transferred to nitrocellulose membranes. After blocking with 5% non-fat milk, the membranes were incubated with the following primary antibodies from Cell Signaling Technologies overnight at 4 °C: phospho-MAPK family antibody sampler kit (#9910), NF-*κ*B pathway sampler kit (#9936), mTOR Substrates Antibody Sampler Kit (#9862) and loading control anti-H3 antibody (#12648, HRP Conjugate). Detection was performed with the provided secondary antibodies using immobilon crescendo Western HRP substrate (#WBLUR0500, Merck, Darmstadt, Germany), and images were captured with either a Fusion-Fx7 spectra reader (Vilber Lourmat, Eberhardzell, Germany) or iBright™ CL1500 system (Thermo Fischer Scientific). Band intensities in Western blots were quantified with ImageJ software (imagej.nih.gov, version: 1.52a, accessed on 1 December 2021) as relative light unit (RLU) normalized to histone H3 loading control.

### 2.7. Fluorescence Labeling, Uptake, and Microscopy

Recombinant proteins were labeled with the Alexa Flour 488 microscale protein labeling kit (Invitrogen) according to the manufacturer’s recommendations. Comparable degrees of fluorescence staining were confirmed by determining the protein concentration based on absorbance at 280 and 494 nm with a NanoDrop ND-1000 (NanoDrop Technologies, Rockland, Del, USA) according to the manufacturer’s recommendations and calculating the degree of labeling (DOL) with the following formula:

DOL = (A_494_ × Dilution factor)/(71,000 × Protein concentration [M])

Protein concentrations after Alexa Flour 488 labeling was confirmed by BCA (micro BCA protein assay kit; Pierce, Rockford, IL, USA). Subsequently, 6.25 × 10^4^ LA-4 cells/mL were stimulated with equimolar amounts of the labeled proteins (rBetv1, rFlaA, rFlaA plus rBetv1, or rFlaA:Betv1) for 15 min at 37 °C either with or without 90 min of pre-stimulation with chloroquine or cytochalasin A and extensively washed with FACS buffer (PBS, 1% BSA, 0.3% sodium azide, and 24 mmol/L EDTA, pH 8.0). Unspecific binding was blocked using incubation of the cells with Fc-Block (eBioscience) for 30 min and then analyzed for protein uptake in LA-4 cells by means of flow cytometry.

For microscopy analysis, LA-4 cells were seeded overnight at 6.25 × 10^4^ cells/mL in 24-well plates. The next day cells were pre-incubated with either cytochalasin A (1 μg/mL) or chloroquine (10 μM) for 90 min and subsequently stimulated with Alexa Fluor 488 labeled proteins for 15 min in DMEM supplemented with 2% FCS. Cells were then washed three times with PBS and fixed for 10 min at room temperature with 4% (*w/v*) paraformaldehyde solution (Thermo Scientific, Dreieich, Germany). After fixation, cells were washed three times with PBS, and stained with 2 μg/mL of the nuclear marker 4′,6-diamidino-2-phenylindole, dilactate (DAPI) (Thermo Scientific) for 5 min and subsequently washed three times with PBS. The uptake of the Alexa Fluor 488 labeled proteins was visualized under a BZ-X800 fluorescence microscope (Keyence, Neu-Isenburg, Germany) at 26× magnification, the addition of the scale bars and merging of figures were achieved using a BZ-X800 analyzer (Keyence, Neu-Isenburg, Germany).

### 2.8. Mice

BALB/c mice (Jackson Laboratories, Bar Harbor, ME, USA) were bred at the animal facility of the Paul-Ehrlich-Institut under specific pathogen-free conditions.

### 2.9. Preparation of LA-4:DC Co-Cultures

mDCs were generated from BALB/c bone marrow as described previously [19]. mDC preparations were shown to be free of CD49b^+^ NK cells, CD3^+^ T cells, and CD19^+^ B cells (data not shown). BALB/c mDCs (5 × 10^5^ cells/well) were cultured with either (I) LA-4 cells (6.25 × 10^4^ cells/well) alone, (II) 1, 10, or 100 µL of supernatant derived from, respectively, stimulated LA-4 cells, or (III) LA-4 cells plus supernatants in 24-well plates (see above). For lung DC isolation, BALB/c mice were killed by CO_2_ and cardiac perfusion was performed by injecting 20 mL PBS before harvesting the lung tissue. Lungs were digested in a DNase/collagenase (Sigma-Aldrich) solution, and DCs were isolated using CD11c (N418) magnetic microbeads (Miltenyi Biotec, Bergisch Gladbach, Germany). CD11c^+^ lung DCs were either cultured alone (1 × 10^5^ cells/well), or co-cultured with LA-4 cells (3.125 × 10^4^ cells/well) in 48-well plates. Cultures were then stimulated with equimolar amounts of either rFlaA + rBetv1 or rFlaA:Betv1 for 24 h. Subsequently, cytokine secretion into culture supernatants was determined by ELISA (see Section 2.4 above).

### 2.10. Statistical Analysis

Statistical analysis was performed with GraphPad Prism v6 to v8 for Mac or Windows using 2-way ANOVA tests with confidence intervals adjusted for multiple comparisons according to either Bonferroni or Turkey. For statistically significant results the following convention was used: *—*p*-value < 0.05, **—*p*-value < 0.01, ***—*p*-value < 0.001.

## 3. Results

### 3.1. rFlaA:Betv1 Induces Chemokine and IL-6 Secretion from LA-4 Epithelial Cells

Initially, LA-4 cells were characterized for their expression of typical epithelial cell surface markers as well as for their expression levels of TLR5 and MHC I (Supplementary Figure S1). LA-4 cells expressed the type II alveolar epithelial markers EpCAM and CEACAM 1, as well as MHC I and TLR5, while only expressing low levels of both mucin 1 and pancytokeratin (Supplementary Figure S1).

**Figure 1:**
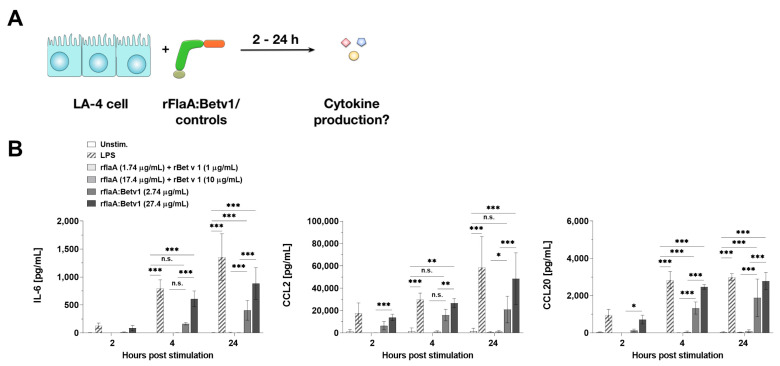
rFlaA:Betv1 induces chemokine and IL-6 secretion from LA-4 epithelial cells. LA-4 cells were stimulated with LPS as a positive control or the indicated equimolar amounts of either rFlaA + rBetv1 or rFlaA:Betv1 for 2, 4, or 24 h (**A**). Supernatants were collected after the indicated time points and checked for the secretion of IL-6, CCL2, and CCL20 by ELISA (**B**). Data are mean results ± SD from three independent experiments with two technical replicates per experiment. Statistical significance indicated as: n.s.: *p*-value > 0.05, * *p*-value < 0.05, ** *p*-value < 0.01, *** *p*-value < 0.001.

In the first set of experiments, LA-4 cells were stimulated with equimolar amounts of either the mixture of rFlaA + rBetv1 or the fusion protein rFlaA:Betv1 for 2, 4, or 24 h, and checked for cytokine and chemokine secretion (Figure 1A).

Compared to the mixture of both proteins, rFlaA:Betv1 induced a significantly increased secretion of the pro-inflammatory cytokine IL-6 as well as the chemokines CCL2 and CCL20 (for the stimulation concentration of 27.4 µg/mL rFlaA:Betv1 induced a 562-fold higher secretion of IL-6, a 37-fold higher secretion of CCL2, and a 30-fold higher secretion of CCL2 24 h post-stimulation) (Figure 1B). IL-6 secretion was detected as early as 4 h post-stimulation, while chemokine secretion was already detected 2 h post-stimulation (Figure 1B).

In this experimental setting no secretion of either GM-CSF, G-CSF, IL-33, TSLP, IL-10, TNF-α, IL-12, or IL-1β was detected from stimulated LA-4 cells (data not shown).

To exclude that the minute amounts of residual LPS contained within the used protein preparations may be responsible for the observed LA-4 activation, both LPS titration curves and stimulations of LA-4 cells were performed applying the amounts of endotoxin contained within the used stimulation concentrations (Supplementary Figure S2). Here, initial LPS-mediated LA-4 cell activation was observed with LPS concentrations around 10 ng/mL (Supplementary Figure S2) which is far above the low pg amounts contained within our protein preparations. Moreover, stimulation of LA-4 cells with the endotoxin amounts contained within the used rFlaA:Betv1 concentrations did not result in secretion of any of the investigated cytokines (Supplementary Figure S2).

Moreover, as previously reported, both rFlaA and rFlaA:Betv1, but not rBetv1, were shown to dose-dependently activate TLR5-expressing HEK293 reporter cells ([22]; data not shown). In addition, rFlaA:Betv1 (or potential contaminations contained within in the preparation) did neither activate TLR2- nor TLR4-expressing HEK293 reporter cells ([35]; data not shown).

### 3.2. rFlaA:Betv1-Mediated LA-4 Cell Activation Is TLR5-Independent

**Figure 2:**
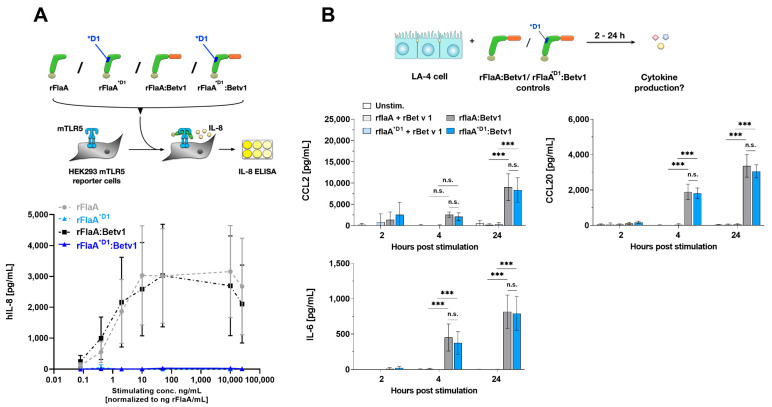
rFlaA:Betv1-mediated LA-4 activation is TLR5-independent. To investigate the TLR5-dependency of rFlaA:Betv1-mediated LA-4 activation, a mutant fusion protein lacking a described TLR5-activation motif was generated (rFlaA^*D1^:Betv1). Wildtype and mutant rFlaA and rFlaA:Betv1 were tested for their capacity to activate TLR5-expressing HEK293 reporter cells (**A**). LA-4 cells were stimulated with protein amounts of both wildtype and mutant rFlaA + rBetv1 and rFlaA:Betv1 equimolar to 10 µg/mL rBetv1 and checked for chemokine and cytokine secretion after the indicated time points post-stimulation (**B**). Data are mean results of three independent experiments ± SD with two technical replicates per experiment. Statistical significance indicated as: n.s. *p*-value > 0.05, *** *p*-value < 0.001.

To address whether the observed LA-4 activation by the fusion protein is TLR5-dependent, we generated a mutant fusion protein that harbors a modified sequence stretch of 8 amino acids (QRMRQLAV on position 87 to 94 replaced with DTVKVKAT) in the flagellin D1 domain (rFlaA^*D1^:Betv1). This sequence alteration was recently described to abolish TLR5 activation [34]. Indeed, both rFlaA^*D1^ and rFlaA^*D1^:Betv1 were unable to activate TLR5-expressing HEK293 reporter cells (Figure 2A).

When LA-4 cells were stimulated with either the wild-type or mutant proteins, we observed no difference in CCL2, CCL20, or IL-6 secretion (Figure 2B), indicating the observed LA-4 activation by rFlaA:Betv1 to be TLR5-independent.

### 3.3. rFlaA:Betv1 Is Taken up More Strongly by LA-4 Cells

**Figure 3:**
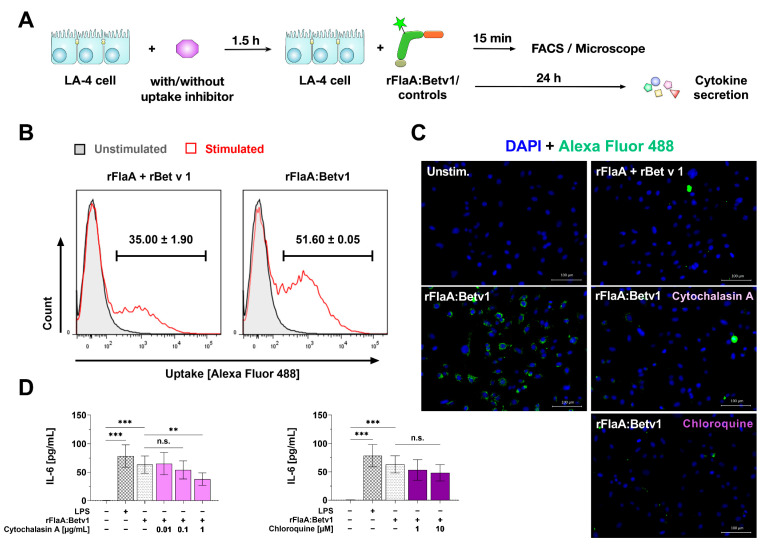
rFlaA:Betv1 is taken up more strongly than the mixture of both single proteins. To investigate the uptake of rFlaA:Betv1, LA-4 cells were stimulated with Alexa Fluor 488 labeled proteins (**A**) and checked for their uptake by either flow cytometry (**B**) or fluorescence microscopy (**C**). In addition, cells were pre-incubated for 90 min with uptake inhibitors and stimulated with the fusion protein for an additional 24 h. The effect of both inhibitors on IL-6 secretion was analyzed 24 h post-stimulation by ELISA (**D**). Data are either representative (**B**,**C**) or mean (**D**) results of three independent experiments ± SD with two technical replicates per experiment. Statistical significance indicated as: n.s.: no statistically significant difference, *p*-value > 0.05, ** *p*-value < 0.01, *** *p*-value < 0.001.

Since the activation of LA-4 cells by rFlaA:Betv1 was shown to be TLR5 independent (Figure 2), we checked if an increased uptake of the fusion protein compared to the equimolar mixture of both single proteins might account for the observed stronger cell activation (Figure 3). For this, we labeled the fusion protein and the respective controls with Alexa Fluor 488 (achieving comparable degrees of labeling) and checked for the uptake of the different proteins into LA-4 cells by either flow cytometry or microscopy (Figure 3A). Here, flow cytometry showed the fusion protein to be taken up with a higher frequency than the equimolar mixture of both proteins (Figure 3B). These results were confirmed by microscopy analysis where we observed extensive uptake of the fusion protein but not the respective controls into LA-4 cells (Figure 3C).

Pre-treatment of LA-4 cells with non-toxic concentrations (Supplementary Figure S3A,B) of the inhibitor of actin polymerization cytochalasin A and the inhibitor of endosomal acidification chloroquine strongly inhibited rFlaA:Betv1 uptake (Figure 3C) and dose-dependently (albeit not significantly for chloroquine) reduced rFlaA:Betv1-induced IL-6 secretion (Figure 3D). In this experimental setting, neither cytochalasin A nor chloroquine affected CCL2 or CCL20 secretion (Supplementary Figure S3C). Controls for the microscopy analysis are shown in Supplementary Figure S4.

### 3.4. rFlaA:Betv1 Induces MAPK- and NF*κ*B-Phosphorylation in LA-4 Epithelial Cells

**Figure 4:**
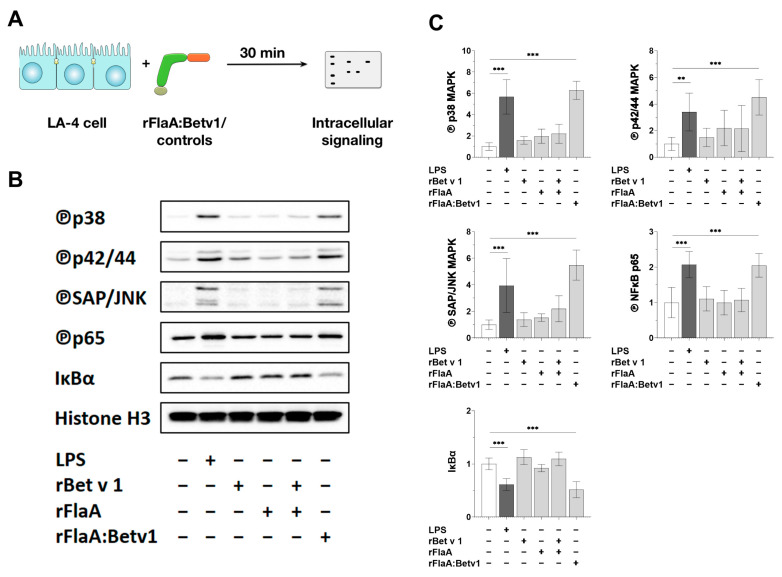
rFlaA:Betv1 induces MAPK- and NF*κ*B-phosphorylation in LA-4 epithelial cells. LA-4 cells were stimulated with either LPS as a positive control, rFlaA, rBetv1, rFlaA + rBetv1, or rFlaA:Betv1 (all equimolar to 10 µg of rBetv1) for 30 min (**A**). Cells were lysed and analyzed by Western blot for the expression levels of the indicated proteins (**B**). The intensity of the Western blot bands from three independent experiments was analyzed and normalized to the expression levels of the loading control histone H3 (**C**). Data are either representative (**B**) or mean (**C**) results from three independent experiments with one lysate generated per experiment. Statistical significance indicated as: ** *p*-value < 0.01, *** *p*-value < 0.001.

To further analyze the intracellular signaling pathways contributing to the observed activation of LA-4 epithelial cells by rFlaA:Betv1, LA-4 cells were stimulated with either LPS, rFlaA, rBetv1, rFlaA + rBetv1, or rFlaA:Betv1 for 30 min, lysed, and analyzed by Western blot (Figure 4A).

Both LPS and rFlaA:Betv1 induced a pronounced phosphorylation of all investigated MAP kinases (p38 MAPK, p42/44 MAPK (also called ERK1/2), and SAP/JNK MAPK) (Figure 4B,C). Moreover, the NF*κ*B subunit p65 was phosphorylated while I*κ*Bα levels were strongly reduced (Figure 4B,C), concordant with an activation of NF*κ*B signaling. In contrast, at equimolar concentrations, the controls rFlaA, rBetv1, and rFlaA + rBetv1 showed no changes in the levels of investigated proteins (Figure 4B,C).

Our previous results from myeloid dendritic cells (mDCs) showed rFlaA:Betv1 to induce both mammalian target of rapamycin (mTOR)-dependent metabolic changes and IL-10 secretion [22]. Therefore, we also checked if mTOR-signaling is activated by rFlaA:Betv1 in epithelial cells. However, there was no pronounced difference in the phosphorylation levels of the mTOR target protein p70 S6 kinase in the different stimulation groups compared to unstimulated controls (Supplementary Figure S5). Moreover, the mTOR inhibitor rapamycin had no significant effect on rFlaA:Betv1-induced cytokine and chemokine secretion (Supplementary Figure S6).

### 3.5. MAP Kinase-Signaling Contributes to Both rFlaA:Betv1-Induced Pro-Inflammatory IL-6 and CCL20 Secretion from LA-4 Cells

**Figure 5:**
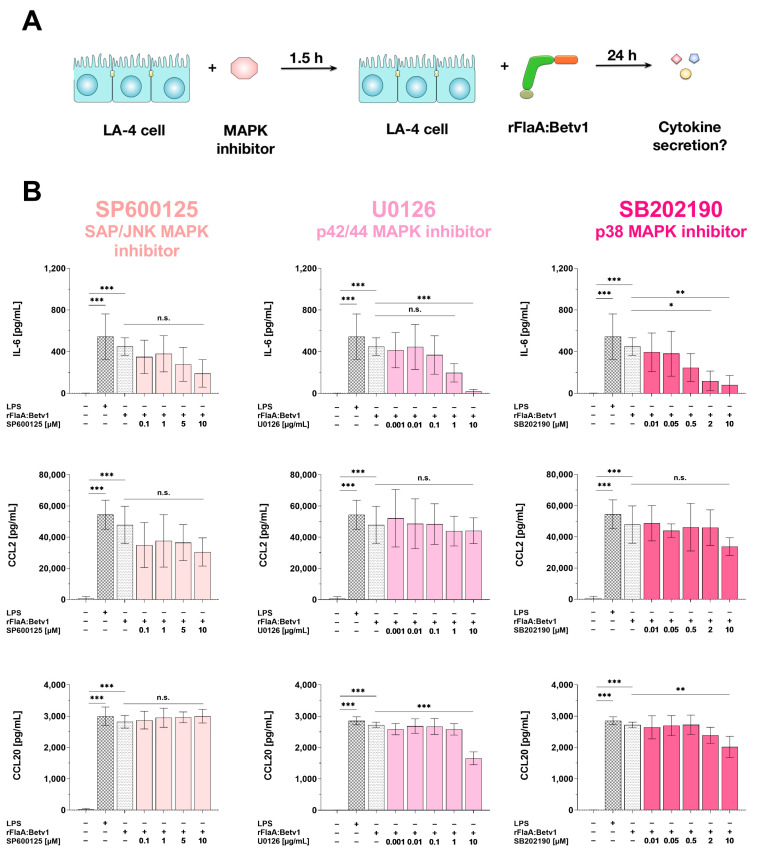
MAPK-signaling contributes to both rFlaA:Betv1-induced pro-inflammatory IL-6 cytokine and chemoattractant CCL20 secretion from LA-4 cells. LA-4 cells were pre-treated with the indicated inhibitor concentrations for 90 min and subsequently stimulated with 27.4 µg/mL rFlaA:Betv1 for 24 h (**A**). Supernatants were collected and checked for the secretion of IL-6, CCL2, and CCL20 by ELISA (**B**). Data are mean results ± SD from three independent experiments with two technical replicates per experiment. Statistical significance indicated as: n.s.: no statistically significant difference, *p*-value > 0.05, * *p*-value < 0.05, ** *p*-value < 0.01, *** *p*-value < 0.001.

To further dissect the contribution of MAPK signaling to the observed IL-6, CCL2, and CCL20 secretion, LA-4 cells were dose-dependently pre-treated with inhibitors of either SAP/JNK MAPK (SP600125), p42/44 MAPK (U0126), or p38 MAPK (SB202190) for 90 min, stimulated with rFlaA:Betv1 for 24 h, and subsequently checked for cytokine and chemokine secretion into the culture supernatants (Figure 5A). Toxic effects of the investigated MAPK inhibitor concentrations on the LA-4 cells were excluded by live/dead staining (Supplementary Figure S7).

All tested MAPK-inhibitors dose-dependently and (except for SP600125) significantly inhibited rFlaA:Betv1-induced IL-6 secretion (Figure 5B). In contrast, CCL2-secretion was unaffected by MAPK inhibition, and CCL20 secretion was only inhibited by high concentrations of U0126 and SB202190, but not by SP600125 (Figure 5B).

### 3.6. NF*κ*B-Signaling Contributes to Both rFlaA:Betv1-Induced Pro-Inflammatory IL-6 and CCL2/CCL20 Chemokine Secretion in LA-4 Cells

**Figure 6:**
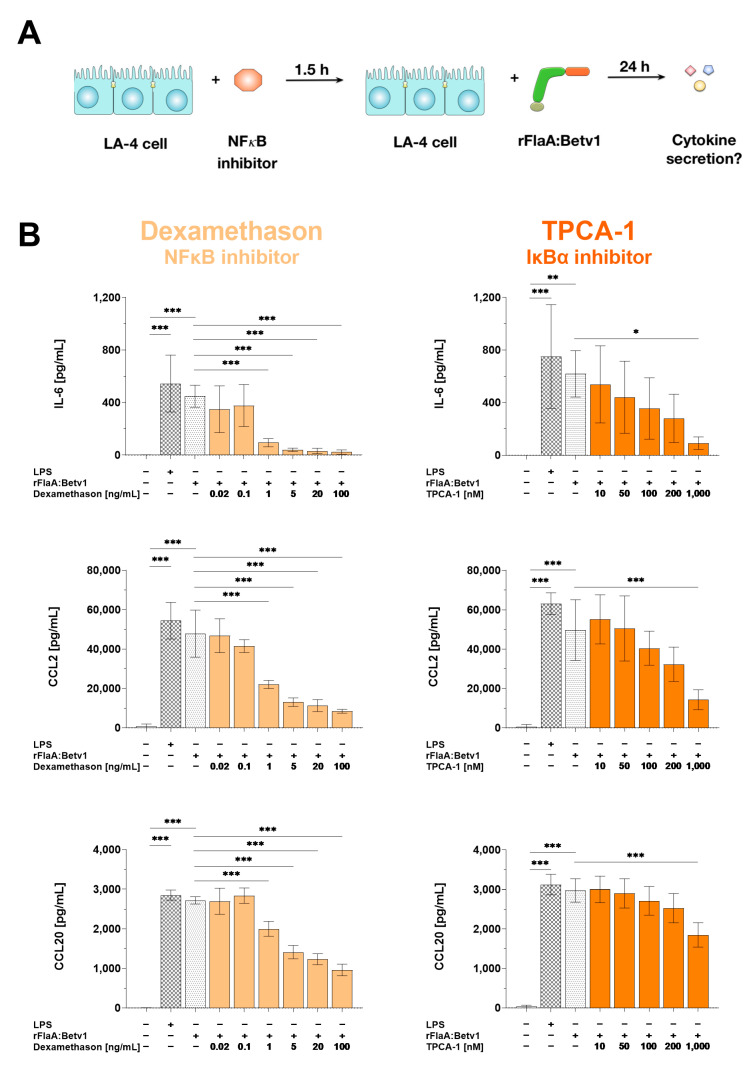
NF*κ*B-signaling contributes to both rFlaA:Betv1-induced pro-inflammatory IL-6 cytokine and CCL2/CCL20 chemokine secretion from LA-4 cells. LA-4 cells were pre-treated with the indicated inhibitor concentrations for 90 min and subsequently stimulated with 27.4 µg/mL rFlaA:Betv1 for 24 h (**A**). Supernatants were collected and checked for the secretion of IL-6, CCL2, and CCL20 by ELISA (**B**). Data are mean results ± SD from three independent experiments with two technical replicates per experiment. Statistical significance indicated as: * *p*-value < 0.05, ** *p*-value < 0.01, *** *p*-value < 0.001.

The contribution of NF*κ*B-signaling to rFlaA:Betv1-mediated cytokine and chemokine secretion was analyzed by pre-treatment of LA-4 cells with either the NF*κ*B-inhibitor dexamethasone or the I*κ*Bα-inhibitor TPCA-1 (Figure 6A). Toxic effects of the applied inhibitor concentrations on the LA-4 cells were excluded by live/dead staining (Figure S8).

Dexamethasone dose-dependently and significantly inhibited rFlaA:Betv1-induced IL-6 (by 95% in the highest inhibitor concentration), CCL2- (by 82%) and CCL20-secretion (by 64%, Figure 6B, left panels), respectively. Similarly, the I*κ*Bα-inhibitor TPCA-1 dose-dependently inhibited rFlaA:Betv1-induced IL-6 (by 85% in the highest inhibitor concentration), CCL2- (by 71%) and to a lower extend CCL20-secretion (by 38%, Figure 6B, middle panels).

### 3.7. In Co-Cultures with BALB/c mDCs LA-4 Cells Modulate mDC-Derived, rFlaA:Betv1-Induced Cytokine Secretion

**Figure 7:**
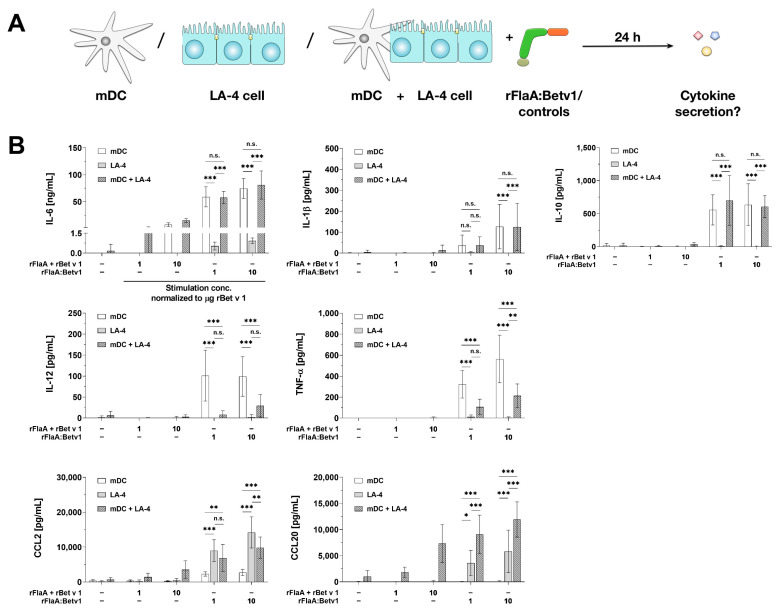
LA-4 cells modulate rFlaA:Betv1-induced cytokine secretion from BALB/c mDCs. BALB/c mDCs and LA-4 cells were cultured either alone or together and stimulated with the indicated equimolar amounts of either rFlaA + rBetv1 or rFlaA:Betv1 (**A**). Cultures were checked for LA-4- and mDC-derived cytokine secretion after 24 h by ELISA (**B**). Data are mean results ± SD from four independent experiments with two technical replicates per experiment. Statistical significance indicated as: n.s.: no statistically significant difference, *p*-value > 0.05, * *p*-value < 0.05, ** *p*-value < 0.01, *** *p*-value < 0.001.

To investigate the functional consequences of rFlaA:Betv1-mediated epithelial cell activation, we checked for the effects of rFlaA:Betv1-stimulated LA-4 cells on mDCs. For this, BALB/c mDCs or LA-4 cells were either cultured alone or as a co-culture, stimulated with rFlaA:Betv1 or their respective controls, and checked for their respective cytokine- and chemokine-secretion (Figure 7A).

Compared to LA-4 cells, mDCs produced significantly higher levels of IL-6 upon stimulation with either rFlaA + rBetv1 or rFlaA:Betv1 in all tested dose ranges (Figure 7B). Here, IL-6 secretion was not significantly different between mDCs cultured alone or together with LA-4 cells (Figure 7B). Moreover, levels of either IL-1β- and IL-10-secretion induced by rFlaA:Betv1 did not differ between mDCs cultured alone or in the presence of LA-4 cells, while LA-4 cells did not produce these cytokines upon stimulation with the tested proteins (Figure 7B).

Secretion of the cytokines IL-12 and TNF-α was exclusively observed from mDCs stimulated with rFlaA:Betv1 (Figure 7B). Interestingly, co-culture of mDCs with LA-4 cells resulted in highly significant reductions in the secretion of both cytokines compared to mDCs stimulated alone (IL-12 reduction by 70% to 92%, TNF-α reduction by 62% to 67%, Figure 7B).

In contrast, the chemokines CCL2 and CCL20 were produced in higher quantities by LA-4 cells (Figure 7B), while mDCs produced a maximum of 2 ng of CCL2 after stimulation with rFlaA:Betv1 and no CCL20 secretion was detected (Figure 7B). While CCL20 levels were consistently higher in co-cultures of mDCs and LA-4 cells compared to LA-4 cells alone, CCL2 levels were slightly decreased upon co-culture of both cell types (Figure 7B).

### 3.8. Epithelial Cell-Derived Soluble Factors Modulate mDC-Responses to rFlaA:Betv1

**Figure 8:**
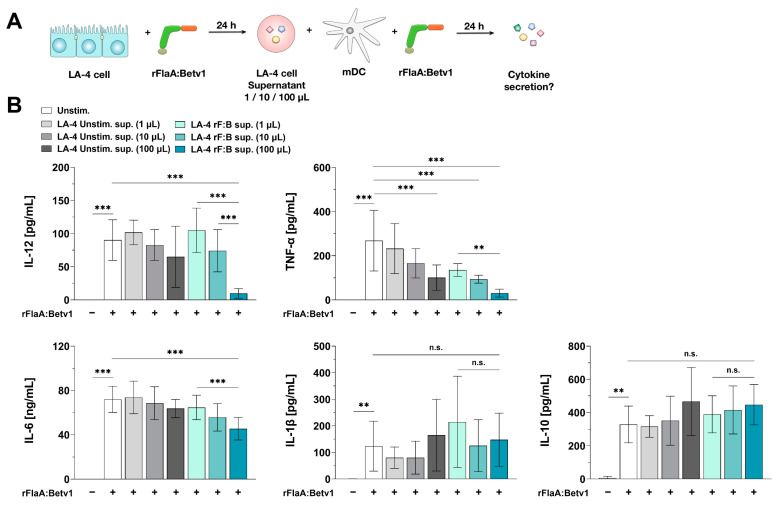
Epithelial cell-derived soluble factors modulate mDC responses to rFlaA:Betv1. BALB/c mDCs stimulated with 27.4 µg/mL rFlaA:Betv1 were incubated for 24 h with 1, 10, or 100 µL of supernatant derived from either unstimulated (LA-4 unstim. sup.) or rFlaA:Betv1-stimulated LA-4 cells (LA-4 rF:B sup.) (**A**). Subsequently, supernatants were collected and checked for the secretion of TNF-α, IL-12, IL-6, IL-1β, and IL-10 by ELISA (**B**). Data are mean results ± SD from three independent experiments with two technical replicates per experiment. Statistical significance indicated as: n.s.: no statistically significant difference, *p*-value > 0.05, ** *p*-value < 0.01, *** *p*-value < 0.001.

We were interested, if the immune-modulating properties of rFlaA:Betv1-stimulated LA-4 cells on mDCs (reduction in IL-12 and TNF-α secretion, see Figure 7B) depend on secreted factors rather than cell-cell contact-dependent mechanisms. Therefore, we incubated mDCs with either 1, 10, or 100 µL of supernatant derived from LA-4 cells that were either unstimulated or stimulated with rFlaA:Betv1. Afterwards, we re-stimulated the mDCs with rFlaA:Betv1 and checked for the effects of LA-4 supernatants on mDC-derived cytokine secretion (Figure 8A).

Supernatant derived from unstimulated LA-4 cells slightly, but not statistically significantly, suppressed rFlaA:Betv1-induced secretion of both IL-12 and IL-6 from mDC cultures, while having no effect on rFlaA:Betv1-induced IL-1β secretion (Figure 8B). In contrast, supernatants of unstimulated LA-4 cells dose-dependently and significantly suppressed rFlaA:Betv1-induced TNF-α secretion (62% reduction for the addition of 100 µL supernatant compared to mDC cultures stimulated with rFlaA:Betv1 in the absence of supernatant) (Figure 8B).

Interestingly, supernatants obtained from rFlaA:Betv1-stimulated LA-4 cells significantly and dose-dependently suppressed rFlaA:Betv1-induced secretion of either IL-6 (by 42%), IL-12 (by 91%), or TNF-α (by 88%) compared to mDC cultures stimulated with rFlaA:Betv1 in the absence of supernatant (Figure 8B). In accordance with the results obtained from mDC:LA-4 co-culture experiments (Figure 7B), LA-4-derived supernatants had no effect on rFlaA:Betv1-induced IL-1β and IL-10 secretion (Figure 8B). These results demonstrate the modulation of mDC responses by rFlaA:Betv1-stimulated LA-4 cells to depend on secreted factors rather than requiring direct cell–cell contact.

### 3.9. The Modulation of mDC Responses by rFlaA:Betv1-Stimulated LA-4 Cells Is Dependent on the p38-MAPK-Signaling Pathway

**Figure 9:**
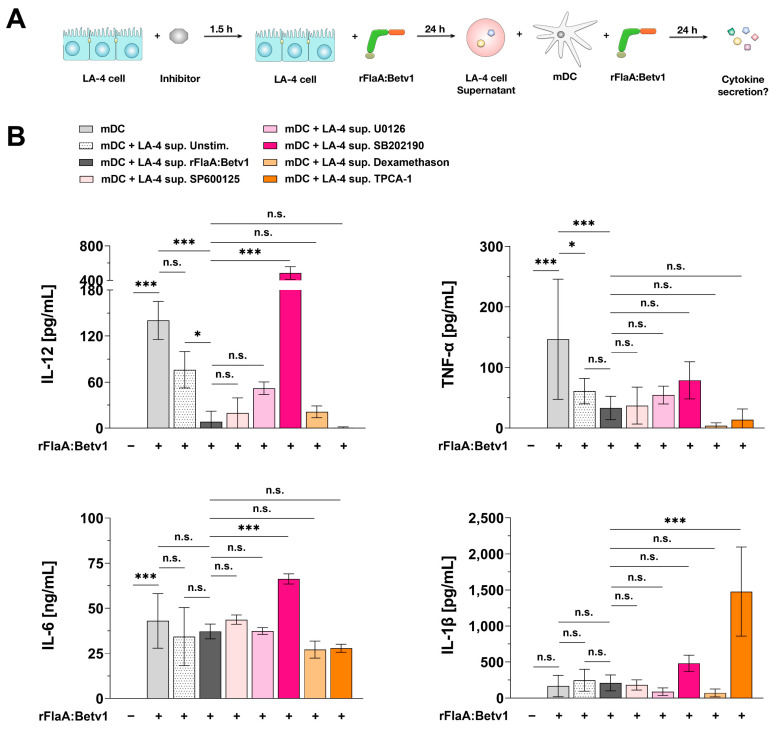
The modulation of mDC responses by rFlaA:Betv1-stimulated LA-4 cells depends on the p38 MAPK-signaling pathway. BALB/c mDCs were either cultured alone or together in the presence or absence of rFlaA:Betv1-re-stimulation. In addition, mDCs were incubated with 100 µL of supernatant (sup.) derived from: (I) unstimulated LA-4 cells (+LA-4 sup. Unstim.), (II) rFlaA:Betv1-stimulated LA-4 cells (+LA-4 sup. rFlaA:Betv1), or (III) LA-4 cells that were pre-treated with the indicated inhibitors for 1.5 h followed by stimulation with 27.4 µg/mL rFlaA:Betv1 for 24 h (+LA-4 sup. (inhibitor name)) (**A**). Supernatants were collected and checked for the secretion of TNF-α, IL-12, IL-6, and IL-1β by ELISA (**B**). Data are mean results ± SD from three independent experiments with two technical replicates per experiment. Statistical significance indicated as: n.s.: no statistically significant difference, *p*-value > 0.05, * *p*-value < 0.05, *** *p*-value < 0.001.

To further investigate the intracellular signaling pathways involved in the production of DC-modulating factors by rFlaA:Betv1-stimulated LA-4 cells, mDCs were treated with supernatants from rFlaA:Betv1-stimulated LA-4 cells that had also been pre-treated with the MAPK- or NF*κ*B-inhibitors previously investigated in Figure 5 and Figure 6 (Figure 9A).

Among the tested supernatants, LA-4 cell-derived supernatants pre-treated with the p38 MAPK-inhibitor SB202190 lost their capacity to suppress mDC-derived cytokine secretion in terms of TNF-α, IL-6, and IL-12 (58.8-fold higher IL-12, 2.4-fold higher TNF-α, and 1.8-fold higher IL-6 levels in mDC + SB202190 sup. vs. rFlaA:Betv1 sup.) (Figure 9B).

In addition to p38 MAPK inhibition, p42/44 MAPK-inhibition by U0126 pre-treatment also slightly reversed the inhibitory effect of rFlaA:Betv1-stimulated LA-4 supernatants on mDC IL-12 production (6.3-fold higher IL-12 levels in mDC + SB202190 sup. vs. rFlaA:Betv1 sup.) (Figure 9B). In this experimental system, neither inhibition of SAP/JNK MAPK by SP600125, NF*κ*B by dexamethasone, nor I*κ*Bα-inhibition by TPCA-1 had a significant effect on the suppression of the tested, mDC-derived cytokines (Figure 9B).

Moreover, mDC-derived, rFlaA:Betv1-induced IL-1β secretion was unaffected by supernatants derived from LA-4 cells treated with either rFlaA:Betv1 or pre-treated with MAPK inhibitors or the NF*κ*B inhibitor dexamethasone (Figure 9B). Here, only I*κ*Bα-inhibition by TPCA-1 pre-treatment resulted in strongly increased IL-1β production (Figure 9B).

### 3.10. Modulation of mDC Responses by rFlaA:Betv1-Stimulated LA-4 Cells Is Dependent on the COX2/PGE_2_ Pathway

Prostaglandin E2 (PGE_2_) is a naturally occurring lipid mediator generated from arachidonic acid. This reaction is catalyzed by either the cyclooxygenase-1 or -2 (COX1/2) enzymes [36]. As an inflammatory mediator, PGE_2_ is also known to influence DC activation, migration, and stimulatory capacity [37].

Previously, epithelial cells were shown to secrete PGE_2_ COX2-dependently upon LPS stimulation [38,39]. Moreover, both PGE_2_ release and COX2 expression were reported to be reduced by the p38-MAPK inhibitor SB202190 upon *Streptococcus pneumoniae* infection in human lung epithelium [40]. Interestingly, PGE_2_ was shown to suppress TLR2- and TLR4-induced IL-12 and TNF-α secretion from both DCs and macrophages in vitro [39,41,42].

**Figure 10:**
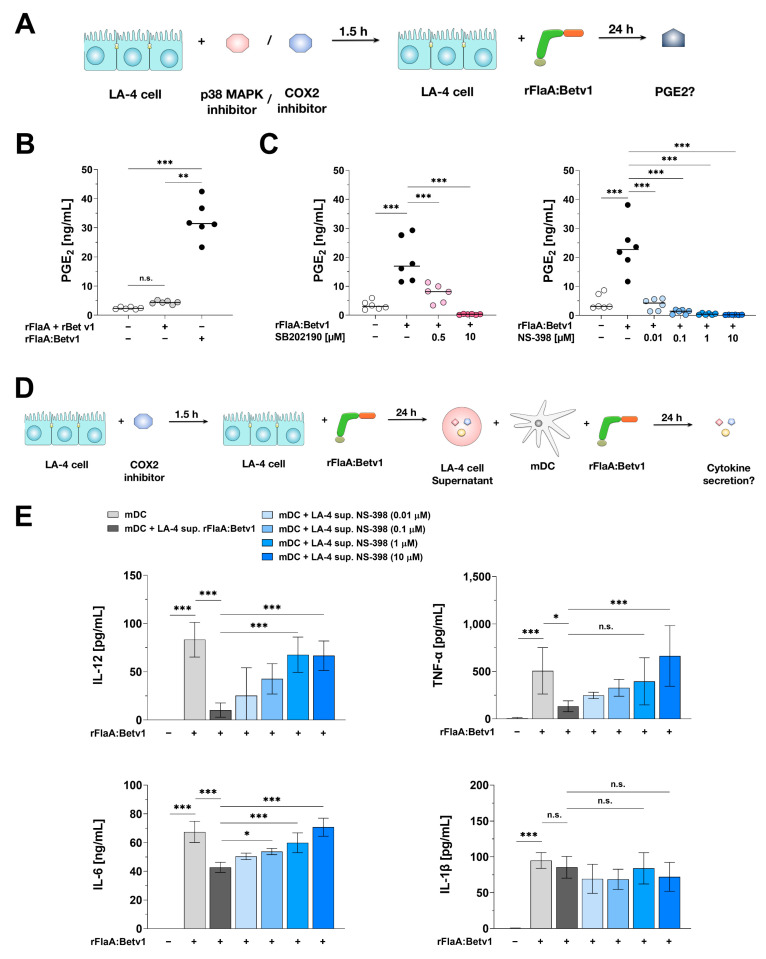
p38-MAPK- and COX2-dependent PGE_2_ production contributes to the modulating effects of rFlaA:Betv1-stimulated LA-4 cells on mDCs. LA-4 cells were pre-treated with either the p38-MAPK inhibitor SB202190 or the COX2 inhibitor NS-398 for 90 min and subsequently stimulated with 27.4 µg/mL rFlaA:Betv1 for 24 h (**A**). Supernatants were collected and checked for the secretion of PGE_2_ by ELISA (**B**,**C**). BALB/c mDCs were cultured in the presence or absence of rFlaA:Betv1-stimulation. In addition, mDCs were incubated with 100 µL of supernatant (sup.) derived from: (I) rFlaA:Betv1-stimulated LA-4 cells (+LA-4 sup. rFlaA:Betv1), or (II) LA-4 cells that were pre-treated with the indicated amounts of the COX2-inhibitor NS-398 for 1.5 h followed by stimulation with 27.4 µg/mL rFlaA:Betv1 for 24 h (+LA-4 sup. NS-398) (**D**). Supernatants were collected and checked for the secretion of TNF-α, IL-12, IL-6, and IL-1β by ELISA (**E**). Data are mean results ± SD from three independent experiments with two technical replicates per experiment. Statistical significance indicated as: n.s.: no statistically significant difference, *p*-value > 0.05, * *p*-value < 0.05, ** *p*-value < 0.01, *** *p*-value < 0.001.

Therefore, we next analyzed the contribution of the p38-MAPK/COX2/PGE_2_ axis to the DC modulating properties of rFlaA:Betv1-stimulated LA-4 cells. We first analyzed if rFlaA:Betv1 could induce p38-MAPK/COX2 dependent PGE_2_ production from LA-4 cells (Figure 10A). Results showed that rFlaA:Betv1 induced a higher PGE_2_ secretion compared to either the unstimulated control (13.4 fold) or the mixture of rFlaA + rBetv1 (7.4 fold) (Figure 10B). Furthermore, both the p38-MAPK inhibitor SB202190 and the COX2 inhibitor NS-398 dose-dependently and significantly inhibited rFlaA:Betv1-induced PGE_2_ release (by 98.5% and 99.2% in the highest inhibitor concentration, respectively) (Figure 10C). We next analyzed the contribution of COX2 to rFlaA:Betv1-induced cytokine and chemokine secretion from LA-4 cells. For this, we pre-treated LA-4 cells with the COX2-inhibitor NS-398 for 90 min, subsequently stimulated with rFlaA:Betv1 for 24 h, and checked for cytokine and chemokine secretion in the supernatant (Supplementary Figure S9A). Toxic effects of the used NS-398 concentrations on LA-4 cells were excluded by live/dead staining (Supplementary Figure S9B). Here, NS-398 dose-dependently inhibited rFlaA:Betv1-induced IL-6 secretion by approx. 30% (Supplementary Figure S9C), but had no effect on chemokine production (Supplementary Figure S9D).

Finally, we incubated mDCs with 100 µL of supernatant derived from LA-4 cells which were pre-treated with increasing concentrations of NS-398, and then checked for mDC-derived cytokine secretion upon re-stimulation with rFlaA:Betv1 (Figure 10D).

Suppression of COX2 in LA-4 was accompanied by an increase of cytokine (IL-12, TNF-α and IL-6) secretion from mDC induced by rFlaA:Betv1 (Figure 10E). Therefore, COX2-inhibition completely reversed the suppressive effect of the supernatant from rFlaA:Betv1-treated LA-4 cells.

## 4. Discussion

In the present study, we analyzed the immune modulating properties of a flagellin:antigen fusion protein on epithelial cells. While the effects of flagellin on epithelial cells are well-known [43,44,45,46,47,48,49,50,51] and the interaction of flagellin with epithelial cells has often been described as a key factor for its adjuvant activity [52], our study is the first to characterize the response of epithelial cells to a flagellin:antigen fusion protein. We could show that, upon stimulation with a flagellin:antigen fusion protein, epithelial cells can modulate pro-inflammatory mDC responses by a both p38 MAPK- and COX2-dependent production of PGE_2_.

Several studies reported flagellin to induce the secretion of chemokines (IL-8, CXCL1, and CCL20) and cytokines (TGF-β1, G-CSF, GM-CSF, and IL-6) from both airway and intestinal epithelial cells [43,44,45,46,47,48,49,50,51]. Mechanistically, flagellin-induced CCL20, TGF-β1, IL-8, and IL-6 production from epithelial cells were shown to be both MAPK- [44,45,50,53] and at least in part NF*κ*B-dependent [51,53,54]. Moreover, Ramirez-Moral et al. found that *Pseudomonas aeruginosa* flagellin induced a mTOR-dependent activation of glycolysis, paralleled by the secretion of CXCL1, CXCL8, CCL20, and G-CSF in primary human bronchial epithelial cells [48]. Interestingly, a recent publication investigated the role of flagellin type C (FliC) from *Salmonella enterica* as a vaccine adjuvant. Here, FliC induced a TLR5-dependent secretion of GM-CSF from airway epithelial cells and further promoted the expansion and activation of type 2 conventional dendritic cells (cDC2s), which led to increased Ag-specific CD4^+^ T cell activation and antibody responses in vivo [47]. These findings prompted us to investigate the interplay between epithelial cell activation and mDC responses to our flagellin:antigen conjugate which is suggested for the intervention of IgE-mediated allergies.

In our study, we observed that compared to the mixture of both proteins, the flagellin:allergen fusion protein rFlaA:Betv1 induced a significantly increased secretion of the myeloid chemo-attractants CCL2 and CCL20 as well as the cytokine IL-6 as early as two hours post-stimulation. This strongly enhanced epithelial cell activation is in accordance with our previous results showing rFlaA:Betv1 to also activate mDCs [22,23] and macrophages [35] more strongly than equimolar amounts of both single proteins. Of note, we observed no secretion of either GM-CSF, G-CSF, IL-33, TSLP, IL-10, TNF-α, IL-12, or IL-1β from LA-4 cells after rFlaA:Betv1-stimulation (data not shown).

**Figure 11:**
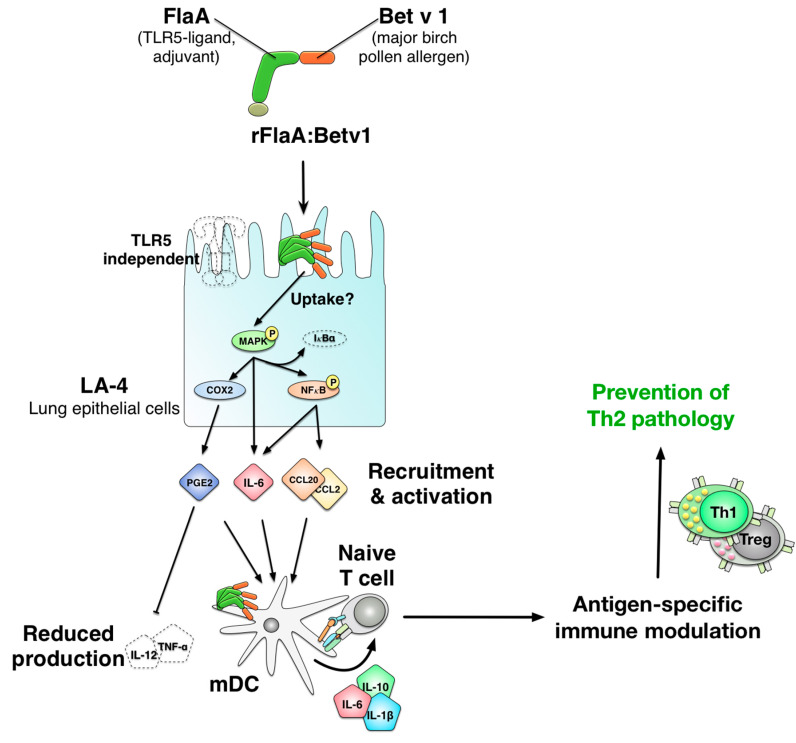
Proposed mechanism of rFlaA:Betv1-mediated LA-4 epithelial cell activation and subsequent modulation of rFlaA:Betv1-induced immune responses by dendritic cells. Stimulation of the mouse lung epithelial cell line LA-4 with the fusion protein rFlaA:Betv1 (combining the TLR-ligand flagellin with the major birch pollen allergen Betv1), resulted in an activation of MAPK- (driving IL-6 secretion), and NF*κ*B-signaling (driving both IL-6 and chemokine secretion). In co-culture with mDCs, LA-4 cell-derived soluble factors/PGE_2_ modulated mDC responses induced via rFlaA:Betv1 by reducing their secretion of the pro-inflammatory cytokines IL-12 and TNF-α. This reduction of IL-12 and TNF-α secretion was dependent on both p38-MAPK-signaling and COX2-mediated PGE_2_ secretion from rFlaA:Betv1-stimulated LA-4 epithelial cells. Therefore, it is possible that modulation of mDC responses by rFlaA:Betv1-stimulated epithelial cells may contribute to the antigen-specific immune-modulatory effects of flagellin:antigen fusion proteins observed before in vivo (prevention of allergen-specific Th2 responses in our case).

Mechanistically, rFlaA:Betv1 was taken up into LA-4 cells more strongly than the mixture of both proteins and rFlaA:Betv1-mediated epithelial cell activation was shown to result from activation of both intracellular MAPK- (p38-, p42/44-, and SAP/JNK-) and NF*κ*B-signaling as inhibition of both pathways prevented cytokine and chemokine secretion. Here, inhibition of NF*κ*B, I*κ*Bα, and all types of MAPK dose-dependently inhibited rFlaA:Betv1-induced IL-6 secretion. Interestingly, rFlaA:Betv1-induced CCL2 and CCL20 secretion were shown to be suppressed by either NF*κ*B or I*κ*Bα inhibition. In contrast, MAPK-signaling did not contribute to the secretion of the investigated chemokines (the suggested molecular mechanism of rFlaA:Betv1-mediated epithelial cell activation is shown in Figure 11). Interestingly, treatment with both proteins alone or as a mixture did not result in comparable MAPK- or NF*κ*B-activation, cytokine-, or chemokine secretion.

Van Maele et al. showed that non-hematopoietic cells stimulated with flagellin to play a key role in TLR5-dependent, flagellin-mediated adjuvant activity by triggering CCL20 secretion from epithelial cells [55]. In line with this, titration rFlaA on LA-4 cells also resulted in the dose-dependent secretion of both CCL2 and CCL20 when applying higher amounts of FlaA (>17.4 µg/mL, data not shown). These results show, that in higher stimulation concentrations, rFlaA can also activate the investigated epithelial cells. However, it needs to be considered that both studies were performed with different subtypes of flagellin.

Using a fusion protein mutant unable to activate TLR5 we showed the rFlaA:Betv1-mediated LA-4 cell activation to be TLR5 independent. Although flagellin is a TLR5 ligand, flagellin may achieve its effects TLR5 independently. In previous studies, TLR5 activation by flagellin was found to be MyD88-, MAPK-, and NF*κ*B-dependent [44,45,50,51,53]. Nevertheless, while some TLR5-expressing epithelial cell lines showed a strong activation after flagellin-stimulation (HT29 and A549), others showed only a weak (HeLa, 293T) or no response (T98G) to flagellin [53]. In line with these results, Tallant et al. suggested that the activation of other factors besides TLR5 could be necessary for a complete response to flagellin [53]. Recent studies indicate, for example, that TLR11, a TLR that is highly expressed in various epithelial cells [56,57], can recognize flagellin TLR5 independently [58]. Interestingly, Hatai and colleagues found that TLR11 only acted as a binding receptor for FliC under acidic conditions (pH 6.0), whereas TLR5 interacted with FliC at pH 6.0 and pH 7.0, suggesting that TLR11 recognizes FliC endolysosomatically [59]. As intracellular processing of the fusion protein was not the focus of this study, further research will be necessary to determine more precisely how flagellin:allergen fusion proteins like rFlaA:Betv1 MAPK-, NF*κ*B-, and COX2-dependently activate epithelial cells after their uptake.

According to previous reports, the stronger LA-4 cell activation by the fusion protein may result from the observed high-molecular aggregation of rFlaA:Betv1 [22], potentially resulting in higher densities of the fusion protein on the cell surface, facilitating protein uptake, and thus stronger activating signals being transmitted to the respective cell’s nucleus. In line with this, inhibition of rFlaA:Betv1 uptake into LA-4 cells reduced fusion protein-induced IL-6 secretion but had no effect on chemokine production. Therefore, we speculate that other, yet unknown mechanisms are likely also engaged in rFlaA:Betv1-mediated epithelial cell activation.

In our previous studies, stimulation of mDCs and macrophages with rFlaA:Betv1 also resulted in an mTOR-dependent metabolic shift towards Warburg metabolism (also called aerobic glycolysis) [22,23,35]. However, in rFlaA:Betv1-stimulated LA-4 cells, we did not observe activation of mTOR signaling. Moreover, the mTOR inhibitor rapamycin had no significant effect on rFlaA:Betv1-induced cytokine and chemokine secretion, suggesting only a minor contribution of increased glucose metabolism to rFlaA:Betv1-mediated epithelial cell activation. These results might be explained by our observation, that LA-4 cells predominantly rely on oxidative phosphorylation for energy generation, while the overall metabolism of both mDCs and macrophages is rather glycolytic (data not shown).

Both CCL2 and CCL20 are myeloid chemo-attractants that alert other immune cells to the site of infection [60,61]. The pronounced activation of and chemokine secretion from rFlaA:Betv1-stimulated LA-4 cells prompted us to investigate, if rFlaA:Betv1-activated LA-4 cells could modulate subsequent immune responses induced by the fusion protein. To investigate the functional consequences of fusion protein-mediated epithelial cell activation, we co-cultured BALB/c mDCs with either LA-4 cells or supernatants derived from rFlaA:Betv1-stimulated LA-4 cell cultures.

Upon co-culture of the two cell types, both rFlaA:Betv1-induced IL-12 and TNF-α secretion from mDCs were reduced while the levels of other mDC-derived cytokines (IL-1β, IL-6, and IL-10) remained unchanged. Furthermore, levels of LA-4 cell-derived CCL2 were slightly decreased upon co-culture of both cell types, suggesting that mDCs might metabolize some of the CCL2 produced by the epithelial cells.

Of note, we also observed a suppression tendency of rFlaA:Betv1-induced TNF-α secretion from *ex vivo*-isolated lung DCs (Supplementary Figure S10), suggesting that our findings can at least in part be transferred to the in vivo situation. Here, lung DCs did not produce IL-12 upon stimulation with rFlaA:Betv1 (Supplementary Figure S10).

To further investigate if the immune modulation by LA-4 cells depends on either direct cell–cell contact or soluble factors, mDCs were incubated with cell-free supernatants of rFlaA:Betv1-stimulated LA-4 cells. Our results showed the suppression of IL-12 and TNF-α from BALB/c mDCs to be dependent on LA-4-derived soluble factors. Interestingly, the lower secretion of rFlaA:Betv1-induced cytokines IL-12 and TNF-α from BALB/c mDCs was paralleled by unchanged levels of IL-1β and IL-10. Taken together, these results suggest, that factors secreted from rFlaA:Betv1-stimulated LA-4 cells can specifically modulate mDC responses.

Since rFlaA:Betv1-induced CCL2, CCL20, and IL-6 secretion from LA-4 cells were not responsible for the modulation of mDC responses, other yet unidentified factors might be engaged. PGE_2_ is a naturally occurring lipid mediator generated by COX2-mediated conversion of arachidonic acid [36]. PGE_2_ is known as an inflammatory mediator that influences DC activation, migration, and stimulatory capacity [37]. In allergy, it was shown to suppress allergic airway responses by inhibiting group 2 innate lymphoid cell (ILC2) activation [62], and decreasing the production of the Th2 cytokine IL-13 [63].

PGE_2_ was reported to be secreted by epithelial cells upon LPS stimulation in a COX2-dependent manner [38,39]. Here, COX2 activation was shown to be located downstream of the p38-MAPK pathway [40,64]. In accordance with the results presented in our study, PGE_2_ was shown to suppress TLR2- and TLR4-induced IL-12 and TNF-α secretion from both DCs and macrophages in vitro [39,41,42].

In our study, rFlaA:Betv1 induced a higher secretion of PGE_2_ from LA-4 cells compared to either unstimulated controls or the mixture of both proteins. Finally, pre-treatment of LA-4 cells with either the COX2-inhibitor NS-398 or the p38 MAPK-inhibitor SB202190 dose-dependently suppressed rFlaA:Betv1-induced PGE_2_ production and significantly reduced the inhibitory capacity of supernatants from FlaA:Betv1-treated LA-4 cells on mDCs. Of note, inhibition of either p42/44 MAPK-, SAP/JNK MAPK-, NF*κ*B-, or I*κ*Bα-activation in LA-4 cells did not have comparable effects, suggesting that these pathways do not contribute to the production of immune-modulating, soluble factors from LA-4 cells.

It is tempting to speculate that COX2 in epithelial cells is either directly or indirectly engaged in the immune modulating capacity of rFlaA:Betv1. The importance of COX2 activation is highlighted by the current discussion if administration of COX2 inhibitors such as ibuprofen around the time of vaccination decreases long-term responses to the COVID-19 vaccines.

In this context, it would be possible that the pronounced anti-allergic effects of different flagellin:allergen fusion proteins observed before [17,18,20,21,22] may at least in part result from a reduced production of pro-inflammatory cytokines (IL-12 and TNF-α) from DCs if these cells are confronted with epithelial cell-secreted factors in flagellin:allergen fusion protein-treated animals. This effect could be further promoted by the unchanged secretion of the anti-inflammatory cytokine IL-10 (Figure 11).

One shortcoming of our study is the exclusive usage of a cell line which may or may not reflect the in vivo situation. Future studies will need to investigate the effects of rFlaA:Betv1 on primary epithelial cells. Moreover, investigating the modulation of rFlaA:Betv1-induced epithelial cell responses in an air-liquid interface setting would be of interest to better understand the effects of the fusion protein, but could not be achieved within the limits of this study.

In summary, we investigated the activation of the mouse lung epithelial cell line LA-4 by a vaccine candidate consisting of the TLR5-ligand flagellin and the major birch pollen allergen Betv1. We observed, that rFlaA:Betv1, but not the mixture of both single proteins, triggered an increased uptake and a MAPK-, NF*κ*B-, and COX2-dependent activation of epithelial cells, characterized by a pronounced secretion of the cytokine IL-6, the myeloid chemo-attractants CCL2 and CCL20, as well as PGE_2_. Furthermore, rFlaA:Betv1-stimulated LA-4 cells modulated the activation of mDCs, resulting in lower secretion of the pro-inflammatory cytokines IL-12 and TNF-α, while the levels of the IL-1β and IL-10 remained unchanged. Our current mechanistic understanding of the events contributing to rFlaA:Betv1-mediated LA-4 activation and its potential consequences for subsequent immune responses induced by rFlaA:Betv1 is depicted in Figure 11.

Finally, the results further establish epithelial cells as important target cells for vaccination approaches and increase our understanding of the mechanisms underlying the strong immune-modulatory effects of flagellin-containing fusion proteins observed in vivo. Our work will likely contribute to a potentially safe and efficient application of such vaccines in the future.

## Data Availability

Not applicable.

## Supplementary Materials

The following are available online at https://www.mdpi.com/article/10.3390/cells10123415/s1, Figure S1: Flow cytometric characterization of the used LA-4 cell line, Figure S2: The amounts of LPS contained within the used rFlaA:Betv1 preparations do not induce LA-4 cells activation, Figure S3. Cytotoxicity and effect on chemokine secretion of the used uptake-inhibitors, Figure S4. rFlaA:Betv1 is taken up more strongly than the mixture of both single proteins, Figure S5: mTOR signaling is not activated by rFlaA:Betv1 in LA-4 cells, Figure S6: mTOR signaling does not contribute to rFlaA:Betv1-induced cytokine and chemokine production from LA-4 cells, Figure S7: Cytotoxicity of the used MAPK-inhibitors on LA-4 cells, Figure S8: Cytotoxicity of the used NF*κ*B-inhibitors on LA-4 cells, Figure S9: Cytotoxicity and effect of the COX2 inhibitor NS-398 on chemokine and IL-6 secretion from LA-4 cells, Figure S10. LA-4 cells also suppress rFlaA:Betv1-induced TNF-α secretion from lung dendritic cells.

## Abbreviations

cDC2s	Type 2 conventional dendritic cells
COX2	Cyclooxygenase-2
FliC	Flagellin type C
ILC2s	Group 2 innate lymphoid cells
MAPK	Mitogen-activated protein kinase
mDCs	Myeloid dendritic cells
MPLA	Monophosphoryl lipid A
mTOR	Mammalian target of rapamycin
NLRC4	NOD-like receptor 4
PAMPs	Pathogen-associated molecular patterns
PGE_2_	Prostaglandin E2
PRRs	Pattern recognition receptors
rBetv1	Recombinant birch pollen allergen Betv1
rFlaA	Recombinant flagellin A from *Listeria monocytogenes*
rFlaA:Betv1	Fusion protein consisting of flagellin A from *Listeria monocytogenes* and the major birch pollen allergen Betv1
RLU	Relative light unit
TLR	Toll-like receptor
