# Supplementary material for: The Fusion Protein rFlaA:Betv1 Modulates DC Responses by a p38-MAPK and COX2-Dependent Secretion of PGE2 from Epithelial Cells
Source: Cells. 2021 Dec 4;10(12):3415. doi: 10.3390/cells10123415 (PMC8700022; doi:10.3390/cells10123415)
Supplement: Supplementary file 1 [file cells-10-03415-s001.zip › cells-1399309 SI.pdf]

cells

MDPI

Article

The Fusion Protein rFlaA:Betv1 Modulates DC Responses by a p38-MAPK and COX2-Dependent Secretion of PGE_2 from Epithelial Cells

Yen-Ju Lin [1], Adam Flaczyk [1,2], Sonja Wolfheimer [1], Alexandra Goretzki [1], Annette Jamin [1], Andrea Wangorsch [1], Stefan Vieths [1], Stephan Scheurer [1] and Stefan Schülke [1,*]

[1] Molecular Allergology, Paul-Ehrlich-Institut, 63225 Langen, Germany; Yen-Ju.Lin@pei.de (Y.-J.L.); aflaczyk@mgh.harvard.edu (A.F.); Sonja.Wolfheimer@pei.de (S.W.); Alexandra.Goretzki@pei.de (A.G.); Annette.Jamin@pei.de (A.J.); Andrea.Wangorsch@pei.de (A.W.); Stefan.Vieths@pei.de (S.V.); Stephan.Scheurer@pei.de (S.S.)

[2] Department of Anesthesia, Critical Care and Pain Medicine, Massachusetts General Hospital, Harvard Medical School, Boston, MA 02114, USA

* Correspondence: stefan.schuelke@pei.de; Tel.: +49-6103-77-5209

Citation: Lin, Y.-J.; Flaczyk, A.; Wolfheimer, S.; Goretzki, A.; Jamin, A.; Wangorsch, A.; Vieths, S.; Scheurer, S.; Schülke, S. The Fusion Protein rFlaA:Betv1 Modulates DC Responses by a p38-MAPK and COX2-Dependent Secretion of PGE_2 from Epithelial Cells. *Cells* **2021**, *10*, 3415. https://doi.org/10.3390/cells10123415

Academic Editors: Constantinos Pitsios and Caterina Chliva

Received: 13 September 2021
Accepted: 2 December 2021
Published: 4 December 2021

Publisher's Note: MDPI stays neutral with regard to jurisdictional claims in published maps and institutional affiliations.

Copyright: © 2021 by the authors. Licensee MDPI, Basel, Switzerland. This article is an open access article distributed under the terms and conditions of the Creative Commons Attribution (CC BY) license (http://creativecommons.org/licenses/by/4.0/).

Supplementary information

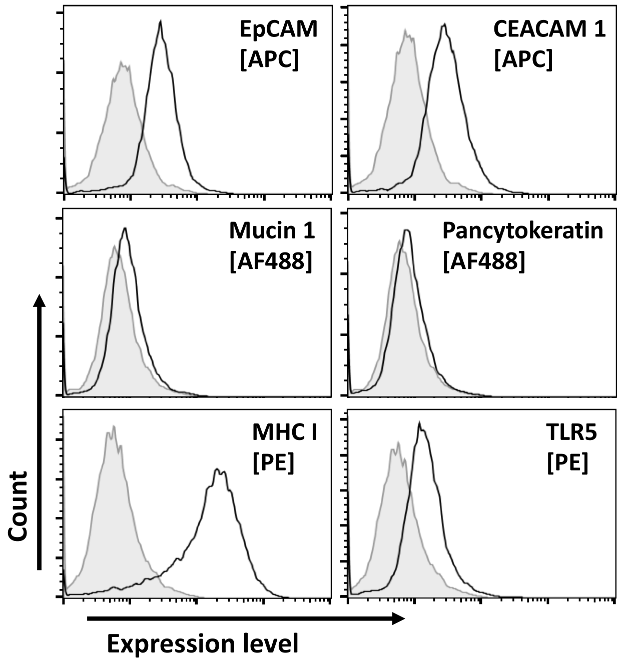

Supplementary Figure S1: Flow cytometric characterization of the used LA-4 cell line. LA-4 cells were stained with the indicated antibodies (black lines) and their expression levels were compared to unstained cells (gray-tinted line). 10,000 cells were measured by flow cytometry using a BD LSR II cytometer. Data are representative results from three independent experiments with 10.000 event recorded per measurement.

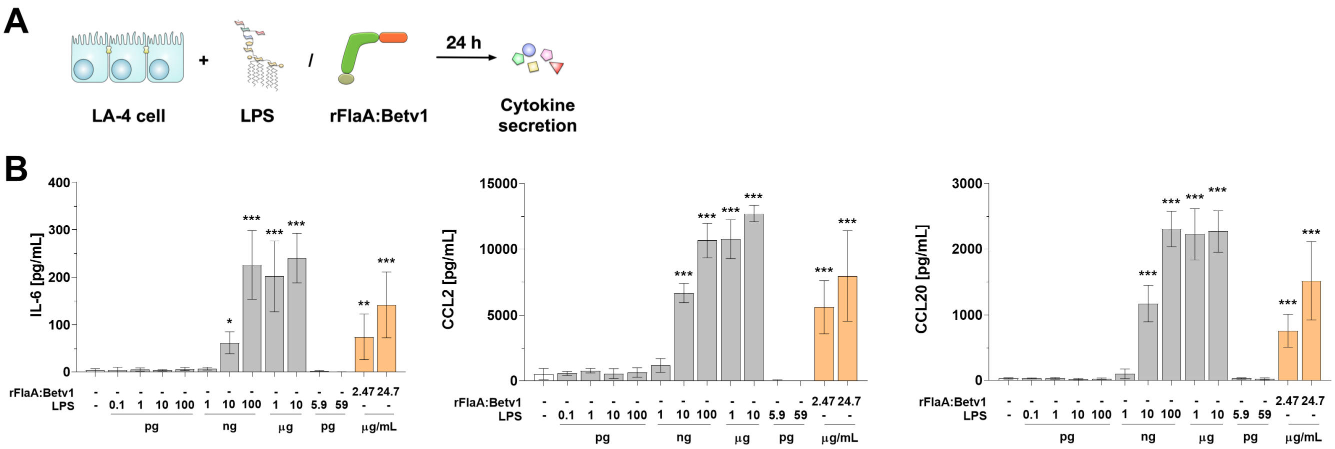

Supplementary Figure S2. The amounts of LPS contained within the used rFlaA:Betv1 preparations do not induce LA-4 cells activation. LA-4 cells were stimulated with either the indicated amounts of LPS to establish dose-response curves (light grey), the residual amounts of LPS contained within the applied concentrations of rFlaA:Betv1 (dark grey), or rFlaA:Betv1 (orange) for 24 h (**A**). Supernatants were analyzed for the induced cytokine and chemokine secretion by ELISA (**B**). Data are mean results of three independent experiments±SD with two technical replicates per experiment.

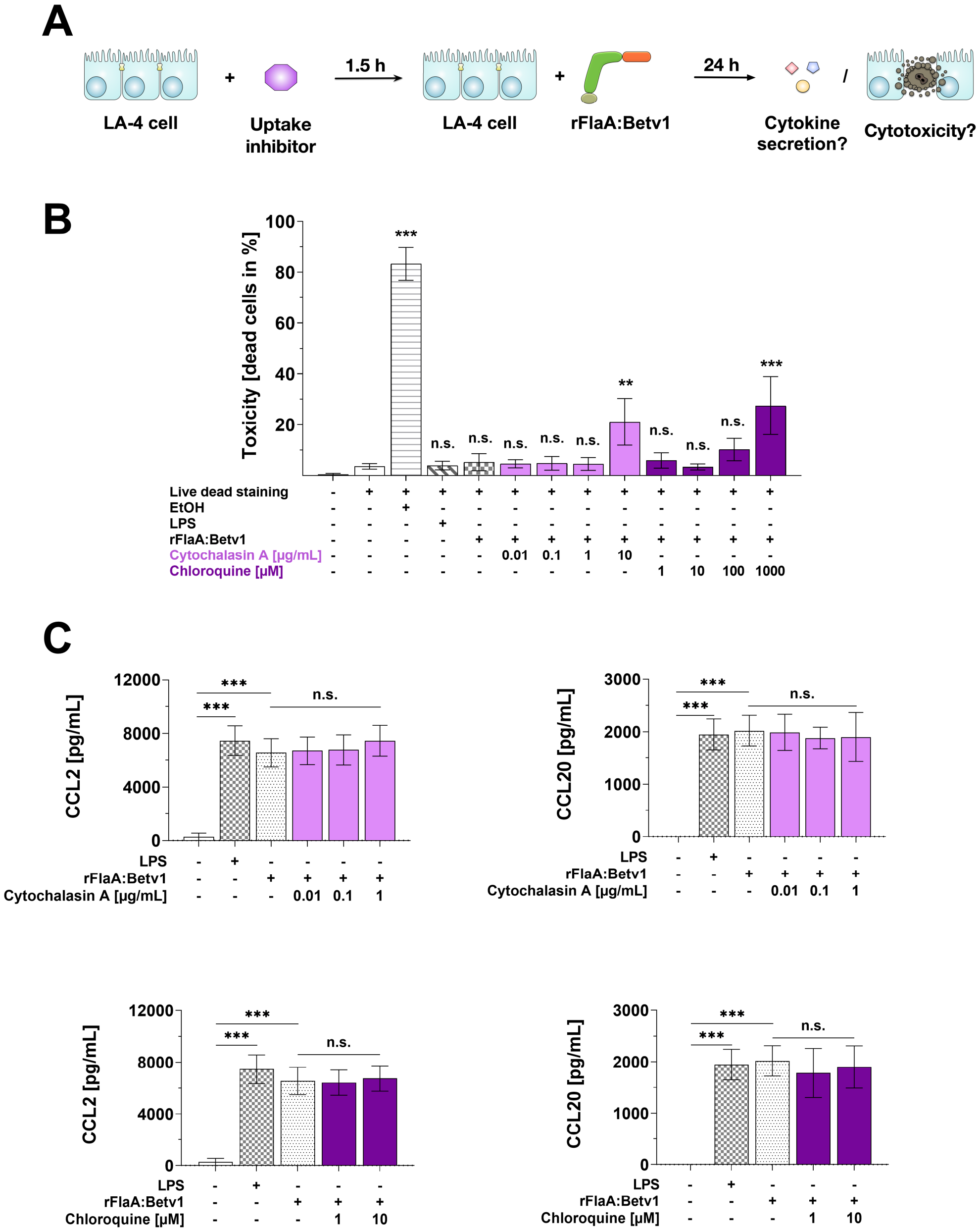

Supplementary Figure S3. Cytotoxicity and effect on chemokine secretion of the used uptake-inhibitors. LA-4 cells were pre-treated with the indicated inhibitor concentrations for 90 min and subsequently stimulated with 27.4 µg/ml rFlaA:Betv1 for 24 h (**A**). Cells killed by incubation for 5 min with 70% ethanol served as positive control. Cells were harvested, stained for dead cells using fixable viability dye, and analyzed for the percentage of dead cells by flow cytometry (**B**). Inhibitor concentrations that showed toxic effects were excluded from the subsequent stimulation experiments. The effect of both inhibitors on chemokine secretion was analyzed 24 h post-stimulation by ELISA (**C**). Data are mean results±SD from three independent experiments with two technical replicates per experiment. Statistical comparisons were performed between indicated samples and unstimulated control samples (**B**) or as indicated (**C**).

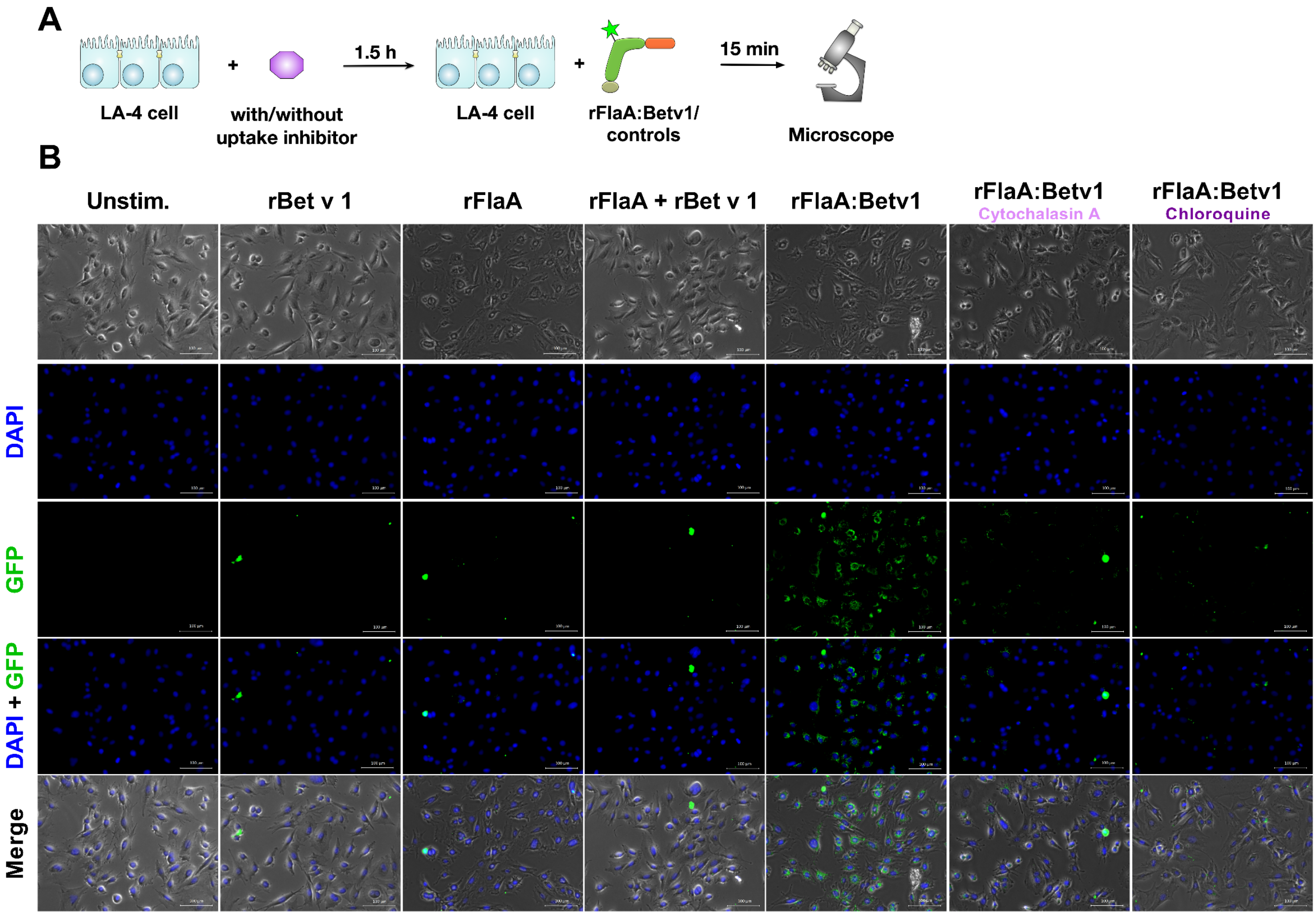

Supplementary Figure S4. rFlaA:Betv1 is taken up more strongly than the mixture of both single proteins. To investigate the uptake of rFlaA:Betv1, LA-4 cells were stimulated with Alexa Flour 488 labeled proteins (**A**) and checked for their uptake by fluorescence microscopy (**B**). In addition, cells were pre-incubated for 90 minutes with the uptake inhibitors cytochalasin A (1 µg/mL) or chloroquine (10 µM) and stimulated with the fusion protein for an additional 15 minutes. Data are representative results of three independent experiments±SD with one technical replicate per experiment.

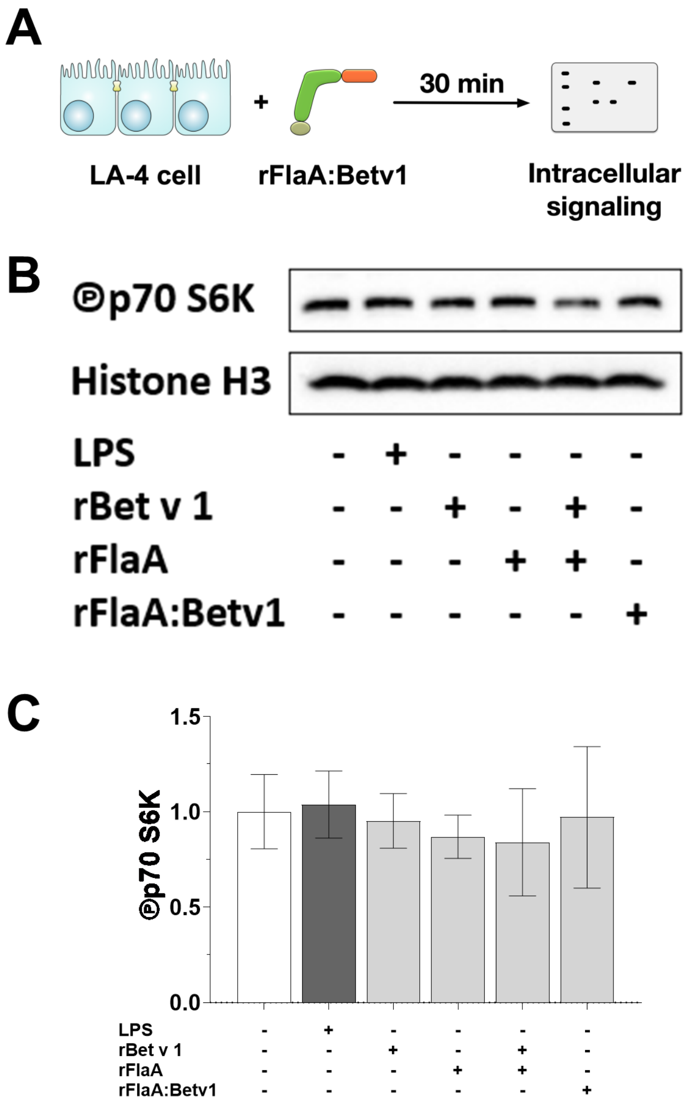

Supplementary Figure S5. mTOR signaling is not activated by rFlaA:Betv1 in LA-4 cells. LA-4 cells were stimulated with either 10 µg LPS as a positive control, rFlaA, rBet v 1, rFlaA + rBet v 1, or rFlaA:Betv1 (all equimolar to 10 µg of rBet v 1) for 30 min (**A**). Cells were lysed and analyzed by Western blot for phosphorylation of the mTOR target protein p70 S6 kinase and expression levels of the internal loading control histone H3 (**B**). The intensities of the Western blot bands were quantified and normalized to the expression levels of the loading control histone H3 (**C**). Data are either representative (**B**) or mean results±SD (**C**) from three independent experiments with one lysate generated per experiment.

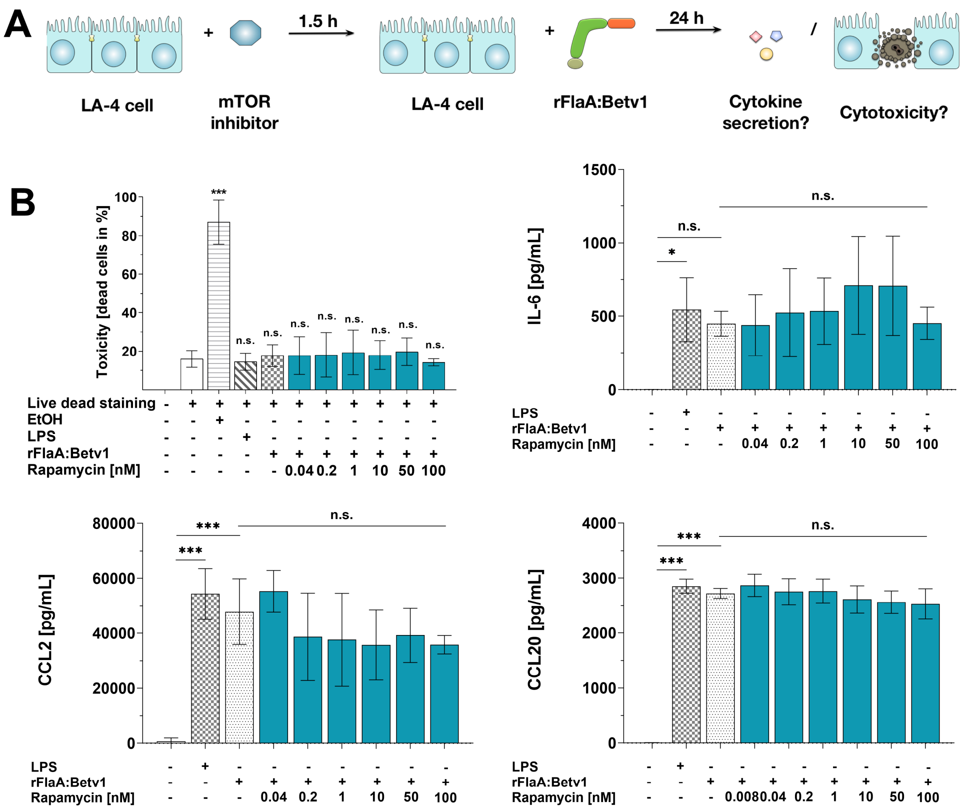

Supplementary Figure S6. mTOR signaling does not contribute to rFlaA:Betv1-induced cytokine and chemokine production from LA-4 cells. LA-4 cells were pre-treated with the mTOR inhibitor rapamycin in the indicated concentrations for 90 min and subsequently stimulated with 27.4 µg/mL rFlaA:Betv1 for 24 h (**A**). Cells were harvested, stained for dead cells using fixable viability dye, and analyzed for the percentage of dead cells by flow cytometry (**B**). As a positive control, cells were killed by incubation with 70% ethanol for 5 min. Furthermore, supernatants were collected and checked for the secretion of IL-6, CCL2, and CCL20 by ELISA (**B**). Data are mean results±SD from three independent experiments with two technical replicates per experiment.

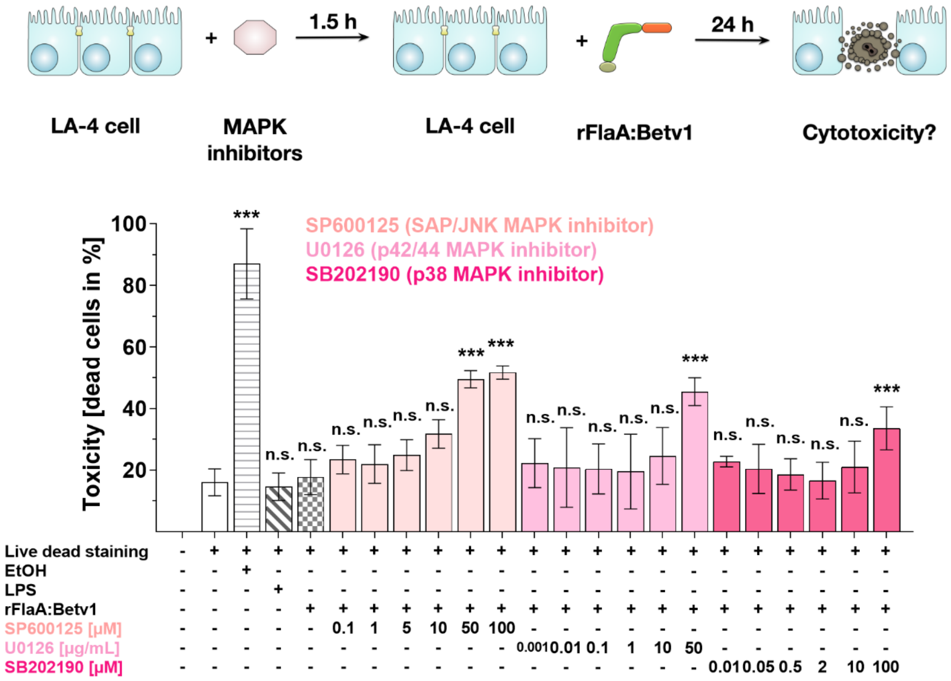

Supplementary Figure S7. Cytotoxicity of the used MAPK-inhibitors on LA-4 cells. LA-4 cells were pre-treated with the indicated inhibitor concentrations for 90 min and subsequently stimulated with 27.4 μg/ml rFlaA:Betv1 for 24 h. Cells killed by incubation for 5 min with 70% ethanol served as positive control. Cells were harvested, stained for dead cells using fixable viability dye, and analyzed for the percentage of dead cells by flow cytometry. Inhibitor concentrations that showed toxic effects were excluded from the subsequent stimulation experiments. Data are mean results±SD from three independent experiments with two technical replicates per experiment. Statistical comparisons were performed between indicated samples and unstimulated control samples.

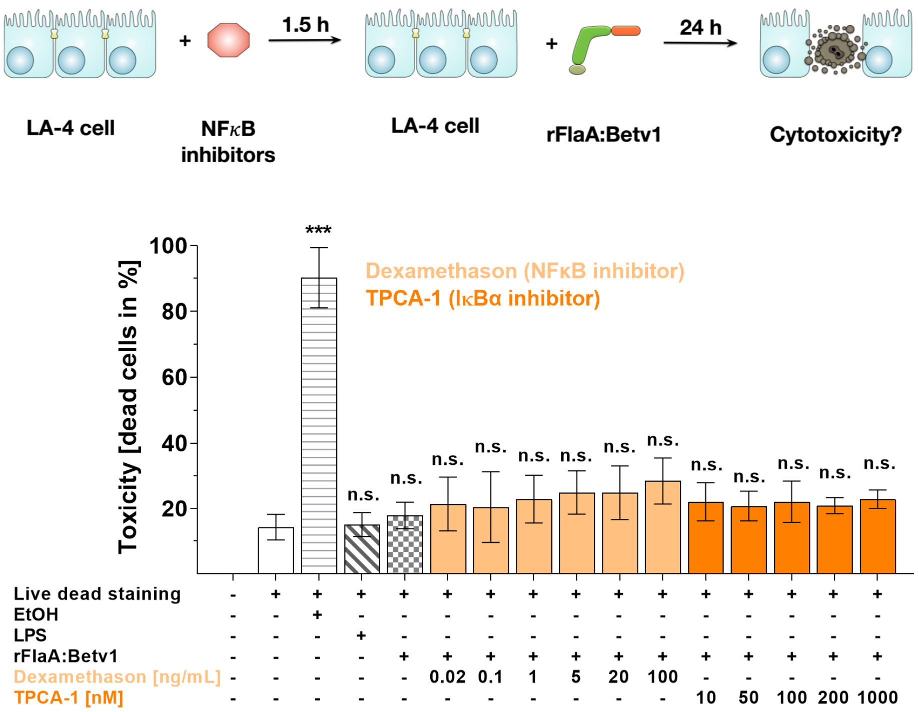

Supplementary Figure S8: Cytotoxicity of the used NFκB-inhibitors on LA-4 cells. LA-4 cells were pre-treated with the indicated inhibitor concentrations for 90 min and subsequently stimulated with 27.4 µg/mL rFlaA:Betv1 for 24 h. Cells killed by 5 min incubation with 70% ethanol served as a positive control. Cells were harvested, stained for dead cells using fixable viability dye, and analyzed for the percentage of dead cells by flow cytometry. Inhibitor concentrations that showed toxic effects were excluded from the subsequent stimulation experiments. Data are mean results±SD from three independent experiments with two technical replicates per experiment. Statistical comparisons were performed between indicated samples and unstimulated control samples.

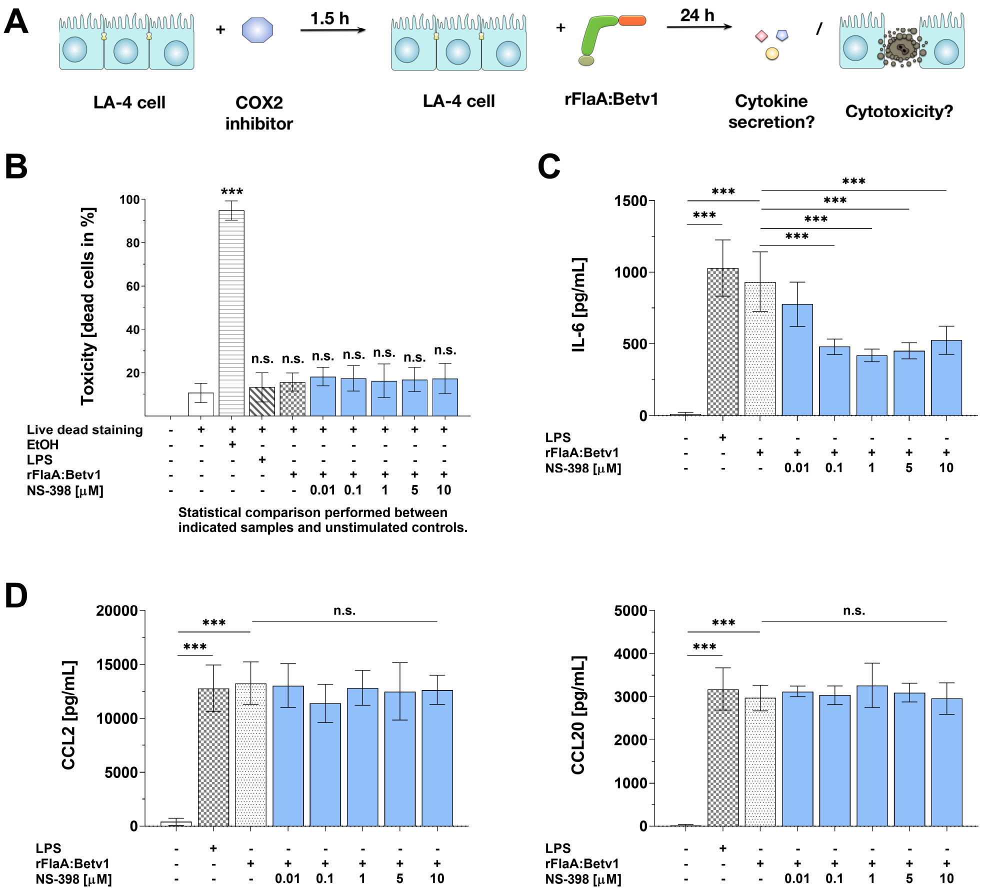

Supplementary Figure S9. Cytotoxicity and effect of the COX2 inhibitor NS-398 on chemokine and IL-6 secretion from LA-4 cells. LA-4 cells were pre-treated with the COX2 inhibitor NS-398 in the indicated concentrations for 90 min and subsequently stimulated with 27.4 µg/mL rFlaA:Betv1 for 24 h (**A**). Cells were harvested, stained for dead cells using fixable viability dye, and analyzed for the percentage of dead cells by flow cytometry (**B**). Supernatants were collected and checked for the secretion of IL-6, CCL2, and CCL20 by ELISA (**C**,**D**). As a positive control, cells were killed by 5 min incubation with 70% ethanol. Data are mean results±SD from three independent experiments with two technical replicates per experiment.

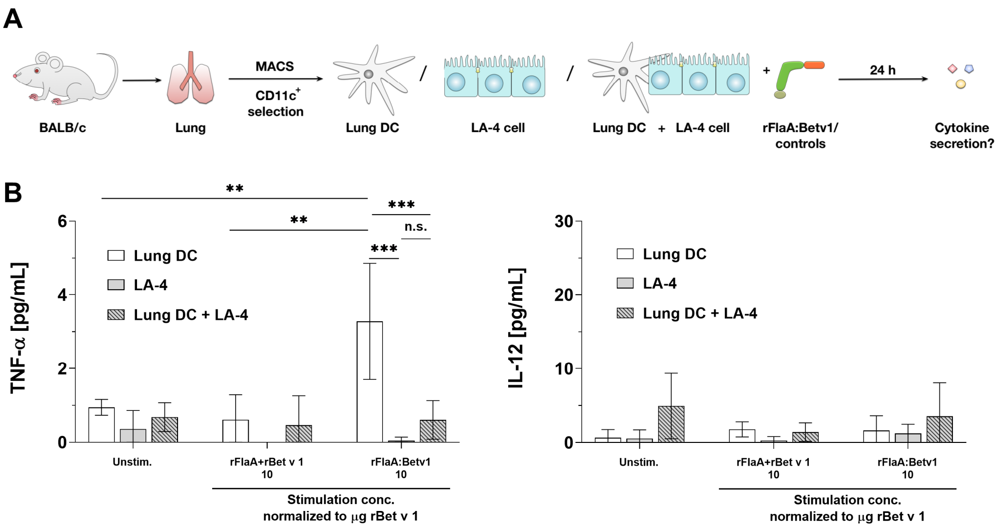

Supplementary Figure S10. LA-4 cells also suppress rFlaA:Betv1-induced TNF-α secretion from lung dendritic cells. Dendritic cells were isolated by magnetic cell sorting from the lungs of naïve BALB/c mice. Lung DCs and LA-4 cells were cultured either alone or together and stimulated with the indicated equimolar amounts of either rFlaA + rBet v 1 or rFlaA:Betv1 (**A**). Cultures were checked for IL-12 and TNF-α secretion after 24 h by ELISA (**B**). Data are results from three independent experiments with one technical replicate per experiment.
